# Model-informed target product profiles of long-acting-injectables for use as seasonal malaria prevention

**DOI:** 10.1371/journal.pgph.0000211

**Published:** 2022-03-14

**Authors:** Lydia Burgert, Theresa Reiker, Monica Golumbeanu, Jörg J. Möhrle, Melissa A. Penny

**Affiliations:** 1 Swiss Tropical and Public Health Institute, Basel, Switzerland; 2 University of Basel, Basel, Switzerland; 3 Medicines for Malaria Venture, Geneva, Switzerland; Institute of Evolutionary Science of Montpellier (ISEM), FRANCE

## Abstract

Seasonal malaria chemoprevention (SMC) has proven highly efficacious in reducing malaria incidence. However, the continued success of SMC is threatened by the spread of resistance against one of its main preventive ingredients, Sulfadoxine-Pyrimethamine (SP), operational challenges in delivery, and incomplete adherence to the regimens. Via a simulation study with an individual-based model of malaria dynamics, we provide quantitative evidence to assess long-acting injectables (LAIs) as potential alternatives to SMC. We explored the predicted impact of a range of novel preventive LAIs as a seasonal prevention tool in children aged three months to five years old during late-stage clinical trials and at implementation. LAIs were co-administered with a blood-stage clearing drug once at the beginning of the transmission season. We found the establishment of non-inferiority of LAIs to standard 3 or 4 rounds of SMC with SP-amodiaquine was challenging in clinical trial stages due to high intervention deployment coverage. However, our analysis of implementation settings where the achievable SMC coverage was much lower, show LAIs with fewer visits per season are potential suitable replacements to SMC. Suitability as a replacement with higher impact is possible if the duration of protection of LAIs covered the duration of the transmission season. Furthermore, optimising LAIs coverage and protective efficacy half-life via simulation analysis in settings with an SMC coverage of 60% revealed important trade-offs between protective efficacy decay and deployment coverage. Our analysis additionally highlights that for seasonal deployment for LAIs, it will be necessary to investigate the protective efficacy decay as early as possible during clinical development to ensure a well-informed candidate selection process.

## 1. Background

Children carry the majority of the global malaria burden, with an estimated 67% of all malaria-related deaths (272 000 in 2019) occurring in those under 5 years of age [1]. In addition to effective and timely treatment, prevention through vector control or drug-based prophylaxis has proven to be an effective approach, reducing incidence and mortality [2]. Especially in seasonal malaria transmission settings, where malaria transmission is linked to the rainy months, well-timed preventive malaria interventions that protect from infection during the transmission months can ease a substantial amount of malaria burden [1]. The WHO has recommended seasonal malaria chemoprevention (SMC) with monthly Sulfadoxine-Pyrimethamine+Amodiaquine (referred to as SMC SP+AQ) for children aged between 3 months and 5 years during the malaria transmission season since 2012 [3]. SP+AQ provides a two-stage effect: while AQ clears existing blood-stage infections, the long clearance half-life of SP prevents new infections. The impact of SMC in seasonal settings has been widely demonstrated [4], achieving a protective efficacy of roughly 80% against clinical episodes in a trial in Burkina Faso [5], a reduction in incidence by 60% in routine implementation in Senegal (80% deployment coverage of all eligible children) [6], and a reduction in the proportion of positive tests by 44% in routine implementation in Mali [7].

Despite its potential, poor adherence limits the effectiveness of SMC and the spread of drug resistance has potential to limit the effectiveness of SMC. Additionally, the monthly delivery of SMC-SP+AQ (one day of SP and three days of AQ) throughout the transmission season is relatively expensive, due to human resources and especially due to operational constraints during the rainy season [8]. Consequently, in 2019, only 62% of children living in SMC-eligible areas in the Sahel subregion received SMC [1]. Throughout the transmission season, coverage subsequently decreased by 6% in Guinea [9] and 20% in Mali [10]. Investigation of the adherence to the three-pill AQ regimen within one treatment course in a clinical trial in Niger showed that only 20% of children received the full regimen [11]. A recent clinical trial reported 93–100% coverage of SMC in Burkina Faso and Mali, which could be considered as part of non-inferiority evaluation for LAIs [12]. Additionally, the spread of resistance markers against SP was reported with increasing SMC deployment [13,14], impacting the eligibility of regions for SMC [15] as well as the protective efficacy after implementation [16].

The need for alternative prevention tools that simplify deployment and possess a reduced risk of resistance is pressing. In the absence of an effective vaccine being deployed, long-acting injectables (LAIs) with an anti-infective effect could provide potential alternative seasonal prevention tools by simplifying the deployment and reducing the risk of resistance through decreased selection pressure for SP resistance [17]. LAIs are still in development and thus far from being used and deployed in malarious areas. Current candidate LAIs include small molecule drugs [18,19] or monoclonal antibodies (mAbs) [20,21] that target the sporozoite stage or liver stage of the malaria parasite, thereby serving as anti-infectives. The successful development of a LAI entails the definition of appropriate product profiles and use cases which are specified in Target Product Profiles (TPPs). Precisely, these specifications include LAI efficacy and safety prerequisites as well as delivery modalities [17]. TPPs are living documents and therefore continuously updated as new evidence for product requirements becomes available.

**Fig 1:**
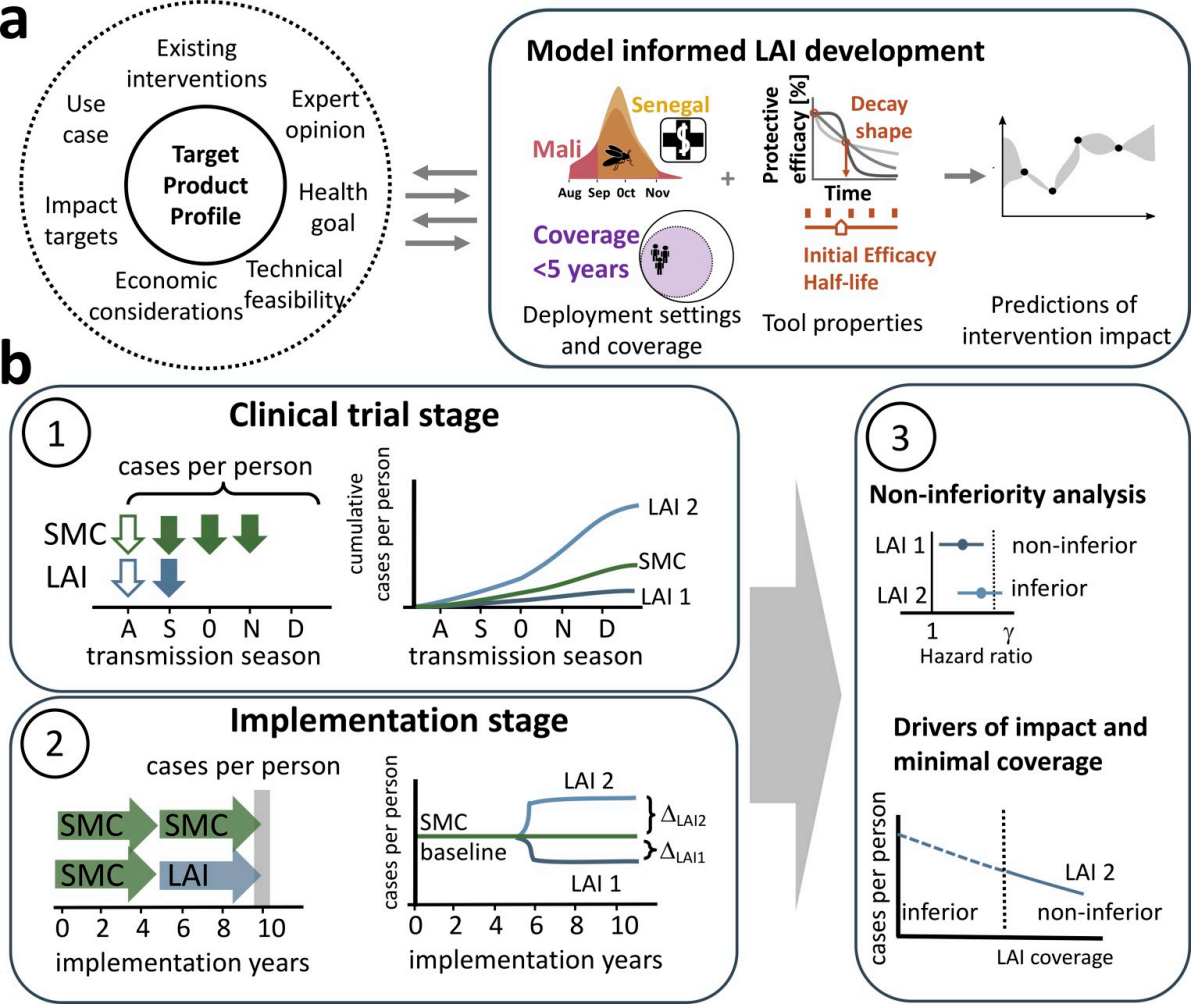
Workflow to assess the target product profile of LAIs. (a) In an iterative exchange between various stakeholders, definition of TPPs is informed by results from modelling approaches. Simulation of predefined scenarios with *OpenMalaria*, an individual-based model of malaria dynamics, allows to predict the likely intervention impact (incidence reduction, indicated by the model symbol) of LAIs in the context of deployment setting details (access to healthcare (indicated by the hospital symbol) and seasonality (indicated by the mosquito icon), deployment coverage of the target population and the tool properties (initial protective efficacy against infection, protective efficacy half-life and decay of protective efficacy). The resulting evaluation of LAI impact is communicated and discussed with stakeholders to refine the analysis as LAIs are developed. (b) The analysis in the clinical trial and implementation stage are illustrated on the example of two hypothetical LAIs with different efficacy profiles (denoted LAI 1 and 2). (1) In clinical trial stages, the minimum essential properties to reach a certain health goal are evaluated in a two-arm clinical trial. SMC with SP+AQ is administered (green arrows) three time during the months of September (S), October (O), and November (N) (as in Senegal, filled green arrows) or four times as well as in August (A) (as in Mali, filled and unfilled green arrows) times over the transmission season. LAIs are administered once at the beginning of the transmission season (blue filled arrow for Senegal or blue empty arrow for Mali). The cumulative cases per person over the trial period are tracked. (2) In implementation stages, SMC-SP+AQ is replaced with LAIs after five years of implementation. Impact is assessed in the last implementation year (grey bar) and compared to the baseline of SMC-SP+AQ implementation. Coverages of SMC-SP+AQ and LAIs are independent from each other. (3) Upper panel: The tool properties influencing the establishment of non-inferiority of LAIs to SMC are investigated in the clinical trial stage. Lower panel: At a fixed SMC deployment coverage, the minimum coverage of LAI deployment required to establish non-inferiority is identified.

To justify the implementation of LAIs under the use case of seasonal malaria prevention, non-inferiority to the existing intervention of SMC-SP+AQ must be met [5]. For new tools with new modes of action and/or deployment modalities, proving non-inferiority to the standard of care is a crucial step and is usually established in non-inferiority clinical trials conducted at late stages of tool development. Currently, it is not yet known what clinical studies will be required for LAIs. In absence of efficacy data on LAIs, it is at the current stage impossible to obtain insights about the circumstances in which LAIs have the potential to be non-inferior. *In silico* modelling and simulation approaches of malaria transmission and control, allow the quantification of the impact of varying tool specifications in relation to varying operational and setting constraints which would not be feasible in real life clinical trials. They thus allow for the exploration of the potential to meet non-inferiority criteria. In the absence of non-inferiority evidence at the early stages of development, modelling and simulation approaches therefore provide a quantitative basis to further inform decision making. They guide tool development from the early stages by predicting potential public health impact (Fig 1A) [22] and understanding non-inferiority criteria prior to clinical trial planning.

Here, we investigate the potential public-health impact for various use cases of LAIs by conducting an *in silico* simulation analysis examining product properties and operational modalities supporting LAI implementation as a seasonal malaria prevention tool. Accordingly, in the simulated scenarios, LAIs were delivered to children under five once at the beginning of the transmission season with an antimalarial in settings currently approved for SMC deployment. Their protective effect was then compared with SMC-SP+AQ administered three or four times per season in monthly intervals. By combining disease modelling and simulation experiments with machine learning approaches, we efficiently explored the large space of possible parameter values describing intervention and transmission setting characteristics and analyzed trade-offs between tool characteristics and operational constraints in a variety of transmission settings [22]. We conducted our malaria transmission simulations using *OpenMalaria*, an established individual-based stochastic simulation platform of malaria transmission and control [23,24]. Based on this approach, we defined a quantitative framework to assess the influence of tool properties and operational constraints on the impact of SMC and LAIs. Using this framework, we investigated two malaria transmission settings based on the malaria transmission profile in Senegal and Mali and assessed public health impact for a plethora of different tool properties, deployment coverages and over multiple transmission intensities. Our analysis was carried out along the clinical development pathway from late clinical trials through to implementation of future LAIs as an SMC replacement. By understanding the main drivers of impact to reach a pre-defined health goal in implementation stages and under operational constraints, we provide an assessment of endpoints in clinical trials of newly developed LAIs and identify efficacy requirements for further development.

## 2. Materials and methods

The impact of novel anti-infective LAIs depends on the tool properties defining their efficacy profile, as well as on the operational constraints and the respective underlying malaria epidemiology, that influence tool suitability for implementation in a given setting (Fig 1A). We investigated the influence of tool properties over a large range of specified protective efficacies, as well as operational considerations (coverage of children) in several implementation settings varying in seasonality and access to healthcare. The drivers of predicted impact for preventive LAIs were analysed along their clinical development from clinical trials to implementation (Fig 1B) and compared with current standard of care (SMC-SP+AQ). Accordingly, we defined two analysis stages: in the *clinical trial stage*, we investigated the maximum incidence reduction and the ability to establish non-inferiority to SMC-SP+AQ over one malaria season (Fig 1B panel 1). In the *implementation stage*, we replaced SMC-SP+AQ with LAIs after five years of implementation at varying coverages, and we inferred the minimal LAI coverage at which LAIs are equivalent (non-inferiority) in public-health impact (incidence reduction) to a continued implementation of SMC-SP+AQ (Fig 1B panel 2).

We adapted a previously developed framework to inform TPPs of new interventions against infectious diseases [22]. First, a set of simulated scenarios was defined. These were characterized by the delivery modality, tool specifications, and settings in which a concrete health target was analysed (in our case, incidence reduction). Second, a set of disease scenarios were simulated randomly over the entire parameter space to evaluate the health outcomes. The resulting database of simulations was used to train a Gaussian process (GP) emulator, which predicts the health outcome given a set of input parameters. Third, the emulator was employed to perform sensitivity analysis and optimisation of tool properties with respect to health outcomes. We employed the use of emulators as it would be computational infeasible to simulate over the entire parameter-space, as well as to perform global-sensitivity analysis and utilize iterative optimization algorithms [25]. This analysis allowed us to define the optimal product characteristics of a LAI that maximises the chance of achieving a desired health goal.

### Malaria disease transmission model

We explored the dynamics of a preventive LAI against malaria using *OpenMalaria*, a stochastic, individual-based simulator of malaria infection in humans linked to a deterministic model of the mosquito feeding cycle [26,27]. *OpenMalaria* accounts for heterogeneity within a human population on multiple levels including host exposure, susceptibility and immune response [23,28,29]. The model allows the investigation of interventions against malaria at multiple points in the malaria life cycle (e.g., vaccination [24], insecticide treated bed-nets [30], and reactive case detection [31]) while monitoring a variety of health outcomes (e.g., prevalence, incidence, and mortality) [32].

### Simulated disease scenarios

Using *OpenMalaria*, we simulated a range of transmission settings (Fig A in S1 Text) and assumptions for the implementation of SMC and LAIs as a seasonal infection prevention intervention replacing existing prevention with SMC-SP+AQ. These assumptions are with regards to the properties of the setting (seasonality and intensity of transmission), health system (access to care and treatment of clinical cases with a first-line ACT), new and replacement intervention (Table 1).

**Table 1:** Summary of simulation set-up used for the implementation experiments.

	Parameter	Setting	Value					
**Health system**	Access to treatment (effective treatment coverage [33] E_14_)	High access	0.5					
		Low access	0.1					
	Diagnostics	-	Rapid diagnostic test					
	Malaria treatment	-	First line: ACT					
		-	Treatment failure/Severe malaria: Quinine					
**Malaria transmission**	EIR [infectious bites per person per year] and yearly cases per person per year [cpppy _0.25-5y_]	High access	EIR	5	9	20	47	150
		High access -long season	cpppy_0.25-5y_	0.42	1	1.6	2.2	2.9
		High access -short season	cpppy_0.25-5y_	0.49	0.95	1.52	2.04	2.83
		Low access	EIR	3	4	8	28	150
		Low access - long season	cpppy_0.25-5y_	0.41	0.71	1.4	2.4	3.3
		Low access - short season	cpppy_0.25-5y_	0.45	0.72	1.3	2.3	3.2
	Mosquito species	*-*	*An*. *gambiae*					
	Biting behavior	*-*	60% indoor biting, 40% outdoor biting					
	Seasonality in malaria transmission [% yearly EIR]		Jul	Aug	Sep	Oct	Nov	Dec
		Senegal-like short season [34]	0	20	60	20	0	0
		Mali-like long season [35]	8	18	50	22	2	0
**Interventions**	Timing of interventions	Senegal-short season	3x SMC: Sep, Oct, and Nov					
		Mali-long season	4x SMC: Aug, Sep, Oct, and Nov					
	Intervention cohort		3mo. – 5 years					
	Population treated		**Coverage: [40 - 100]%**					
	Protective efficacy LAI		**Initial efficacy: [70 - 100]%**					
			**Half-life: [30-150] days**					
			Decay shape:					
		exponential LAIs	Exponential decay, k=1 (Eq 1)					
		biphasic LAIs	Biphasic decay, k=0.69 (Eq 1)					
		sigmoidal LAIs	Sigmoidal decay, k= 8 (Eq 2)					
	Protective efficacy SMC-SP+AQ		Initial efficacy: 100%					
			Half-life: 32 days					
			Decay shape: Weibull, k= 5.4 (Eq 1)					
			Coverage declining by 7-10% over the season					

The simulated transmission settings were defined using a factorial design covering all possible combinations of discrete health system and vector specifications. The parameters defining the efficacy and delivery profiles of LAIs (highlighted in bold in the third column) were sampled within the defined parameter space using Latin Hypercube Sampling and simulated for each combination of settings. The effective coverage E_14_ describes the probability that effective malaria treatment will occur within a 14-day period since symptoms onset. Additional information on simulated transmission intensity can be found in S1 Text (Fig A and Table A in S1 Text).

The intervention age-group consisted of children between 3 months and 5 years of age (accounting for ca. 16% of the total population). The intervention age-group was chosen according to WHO recommendations [3], although some countries have implemented SMC for children up to 10-years-old [36]. We assumed a total population of 10,000 individuals to capture transmission settings within health facility catchment areas with an age-structure that represents a realistic age-distribution for African malaria-endemic settings [37]. Access to treatment, defined as the percentage of the whole population who seek care for a symptomatic malaria episode, was chosen to represent settings with low or high health systems strength. The probability of symptomatic cases (mild or severe) to receive effective treatment within two weeks from the onset of symptoms (E_14_) was set to 10% in low access to healthcare settings and 50% in high access to healthcare settings [38]. The malaria seasonality profile, mosquito species and timing of interventions were parameterised to reflect those of Mali or Senegal, two countries in the Sahel region where SMC is implemented and clinical trials for malaria interventions are conducted frequently. In Mali, the malaria season is longer, starting in August and lasting until November (*long season*), and SMC is generally four monthly doses. In contrast, the malaria season in Senegal is only three months long, with a sharper profile (*short season*) and SMC is three doses one month apart (Fig 1, Table 1, and Fig B in S1 Text). Malaria prevalence in Senegal is generally low, with the highest *P*. *falciparum* prevalence in 2–10 years old (*Pf*PR_2-10y_) in the southern regions being around 8%. However, the *Pf*PR_2-10y_ in Mali is around 80% in the south of Mali but very low in the North [39].

To develop a broader understanding of the influence of transmission intensity on LAI impact, we simulated a range of initial incidence settings capturing the transmission heterogeneity of these two malaria-endemic countries (Fig A and Table A in S1 Text). The simulated transmission intensity of each setting was defined by the entomological inoculation rate (EIR) modelled as the average annual number of infectious bites received by an individual, and the corresponding simulated *Pf*PR_2-10y_ (Table A and Fig A in S1 Text). The protective efficacy of SMC-SP+AQ was implemented as being fully effective (no prevalent resistance against SP) or reduced duration of protection (prevalent resistance against SP).

Over all simulation experiments, the input parameter space for the protective efficacy and its decay, and the intervention coverage are as defined in Table 1. Parameters were randomly sampled using Latin hypercube sampling [40] by drawing 1500 parameter sets for each setting (capturing combinations of seasonality, health system access, EIR, and SP resistance), with 5 stochastic realizations (simulation replicates) for each point. All simulations were performed using *OpenMalaria* version 38. The source code and comprehensive documentation for *OpenMalaria*, including a detailed model of demography, transmission dynamics and interventions is available online [41] or in a recent publication [25].

### Delivery: Clinical trial and implementation assumptions

In our study, LAIs were implemented as anti-infective entities in the form of mAbs or small molecule drugs that prevent the development of blood-stage malaria through action on malaria parasites in sporozoite or liver stages. We assumed administration once at the beginning of the transmission season in combination with an artemisinin-based combination therapy (ACT) that cures prevalent blood-stage malaria infections with a 100% cure rate noting this was an optimistic assumption for ACTs. The same initial blood-stage clearance was applied for SMC-SP+AQ to focus on the investigation of protective efficacy. SMC-SP+AQ was administered monthly, administered for three months per season in Senegal-like settings *(short season*) and for four months per season in Mali-like settings (*long season*) with four seasonal cycles per year for each setting as recommended by the WHO [3] (Fig B in S1 Text). We assume a decrease in SMC coverage by 3–4% between treatments (7% over the three and 10% over the four month season for the treatment regimen), therefore lying between the two extremes of observed coverage decrease [9,10].

The *clinical trial stage* (Fig 1B panel 1) was simulated in the high case management setting to account for awareness of malaria symptoms. Initial deployment coverage levels for both, SMC and LAI, were set to 100%. Follow up visits, in the form of active case detection carried out by health workers at the community and household level for groups considered to be at high risk, were implemented two weeks after every administration round of SMC-SP+AQ in both trial arm. In addition to fully effective SMC-SP+AQ, we investigated a reduced length of protection by prevalent resistance against SP in the *clinical trial stage*.

In the *implementation stages* (Fig 1B panel 2), we analysed the impact of switching seasonal malaria prevention strategies from SMC-SP+AQ after five years of implementation to LAIs. After five years of LAI implementation, the cumulative clinical case incidence was compared with a control setting (no switching). LAIs and SMC-SP+AQ were implemented with varying coverages between 40–100%. Intervention cohorts, defined by the coverage in the intervention age group, were specified at the beginning of a transmission season for the whole season. An exemplary illustration of LAI and SMC-SP+AQ implementation is provided in Fig D in S1 Text.

### Intervention characteristics: SMC and LAI properties

As the decay of efficacy of LAIs is not yet known, we explored a range of possible scenarios. In both the clinical and implementation analysis, the prevention of infection by LAI was modelled as pre-erythrocytic protection from infection *E(t)* over time defined by the initial protective efficacy *E*_*0*_ [%], protective efficacy half-life of decay *L* and shape parameter *k* (Fig C in S1 Text). The decay shapes of protective efficacy were chosen such that they represent multiple development possibilities: exponential-like decay (referred to as *exponential LAIs*), malaria vaccine-like decay, namely biphasic-like decay [42,43] (referred to as *biphasic LAIs*) and sigmoidal-like decay (referred to as *sigmoidal LAIs)*. The protective efficacy decay over time *E(t)* for exponential and biphasic LAIs was modelled as either Weibull-like decay:

$$E(t)=E_{0}\;e^{-{\left(\frac{t}{L}\right)}^{k}\;log\;(2)},$$ Eq 1

where *k* = 1 yields *exponential LAIs* and *k* = 0.69 yields *biphasic LAIs*. *Sigmoidal LAIs* were defined by the following Hill equation with *k* = 8.


$$E(t)=E_{0}\frac{1}{1+{\left(\frac{t}{L}\right)}^{k}}.$$ Eq 2

The individual protection profile over time after one administration of SMC-SP+AQ, was parameterized using published clinical trial results [5]. The protection profile in preventing infections is not well understood [44], and usually the protective efficacy of SMC-SP+AQ was assessed in clinical studies in terms of population survival estimates, risk reductions or a reduction in incidence [10,45]. To compare the impact of novel LAIs with SMC-SP+AQ, we parameterized the protective efficacy of SMC–SP+AQ to data from a clinical non-inferiority trial conducted in Burkina Faso [5]. Our parameterization approach employs a Gaussian process regression model [46] to determine the model parameters that reproduce the clinical trial data via minimization of the residual sum of squares between the observed and simulated SMC-SP+AQ protective efficacy. A detailed description of the parameterization process is given in S1 Text. Scenarios assuming SP resistance, the effect of resistance against the SMC component SP was implemented by decreasing the SMC half-life of protection from 32 to 20 days. The lower protection half-life due to resistance can be modeled as an increase in the drug concentration inhibiting the parasite growth by 50% (IC_50_). Because SP has a long clearance half-life [47], we assume no impact of resistance on the initial efficacy.

### Health target: Endpoints to assess impact of LAI

The health targets analysed in this study were based on incidence reduction by LAIs, clinical cases averted by LAIs compared with SMC, and non-inferiority with regard to clinical burden in the modelled clinical trials. Clinical cases per person (cpp) in the intervention age group (*cpp*_*0*.*25-5y*, *int*_) over the clinical trial length (Fig 1A) (*cpp*_*0*.*25-5y*, *int*_) or in the last implementation year only as cases per person per year (cpppy) (*cpppy*_*0*.*25-5y*, *int*_) (Fig 1B) were calculated over the entire population at risk in the intervention age group (*N*_*0*.*25-5y*, *int*_).


$${cpp}_{0.25-5y,int}=\frac{total\;cases}{N_{0.25-5y,int}}$$ Eq 3

The incidence reduction percentage *inc*_*red*_ was calculated via the cumulative incidence in the year before trial implementation *cpp*_*cont*_ and the cumulative incidence during the clinical trial *cpp*_*int*_

$${inc}_{red}=\frac{{cpp}_{0.25-5y,cont}-{cpp}_{0.25-5y,int}}{{cpp}_{0.25-5y,cont}}\times 100$$ Eq 4

Survival analysis on the number of clinical cases per person *cpp*_*int*_ was performed to analyse the establishment of non-inferiority of LAIs to SMC-SP+AQ as described in [48]. The impact of a LAI is assumed to be non-inferior to SMC-SP+AQ if the upper bound of the 95% confidence interval of the hazard ratio between SMC-SP+AQ and LAI is less than the upper bound for non-inferiority. This upper bound is informed by the survival estimate of SMC-SP+AQ and the desired margin for non-inferiority. We assumed a 5% margin for non-inferiority. More information about the non-inferiority analysis is provided in S1 Text.

Additionally, the intervention impact defined as clinical incidence difference between SMC-SP+AQ clinical cases per person per year *cpp*_*0*.*25-5y*_, _*SMC*_ and LAIs *cpp*_*0*.*25-5y*_, _*LAI*_ was compared using the relative difference *diff*_*cpp*_ as an indicator for the malaria burden.


$${diff}_{cpp}=\frac{{cpp}_{0.25-5y,SMC}-{cpp}_{0.25-5y,LAI}}{{cpp}_{0.25-5y,SMC}}$$ Eq 5

### Gaussian process emulator approach to predict intervention impact

To perform a fast and efficient search of the parameter space of LAI properties, we used a database of *OpenMalaria* simulations to train heteroskedastic GPs for each LAI efficacy decay type in each seasonal and transmission intensity setting (R-package *hetGP*, function *mleHetGP*, Version 1.1.2.) [49]. The input parameters of the GPs in the *clinical trial stage* consisted of the tool properties including initial protective efficacy and protective efficacy half-life. In the *implementation stage*, the input parameters additionally included the respective SMC-SP+AQ and LAI deployment coverage. To define the covariance structure of the Gaussian process models, we used a Matérn kernel with smoothness parameter 5/2 accounting for the high variability in the parameter space [49] The trained GPs were then used to predict the number of cases per person per year and the metrics related to the non-inferiority analysis (Table B in S1 Text) for any point in the parameter space. Emulator performance was ascertained by testing on a 20% holdout set after training on 80% of the data [22] (Table B in S1 Text).

### Sensitivity analysis

We identified the main drivers of intervention impact via a global sensitivity analysis, performed using decomposition of variance via Sobol analysis on the emulator output predictions. We conducted an EIR-stratified sensitivity analysis to assess the potential change in drivers of impact over the whole transmission range. Within the pre-defined parameter space, the total effect indices quantify the interactions between individual parameter contributions to the emulator output variance [50]. The total effect indices were normalized to obtain the relative importance of each parameter through division by their sum. The total effect indices were estimated with a Monte Carlo sampling approach using the function *soboljansen* in the R-packages *sensitivity* (version 1.16.2) with 500 000 sampled points and 1000 bootstrap replicates.

### Intervention properties and coverage optimisation

**Fig 2:**
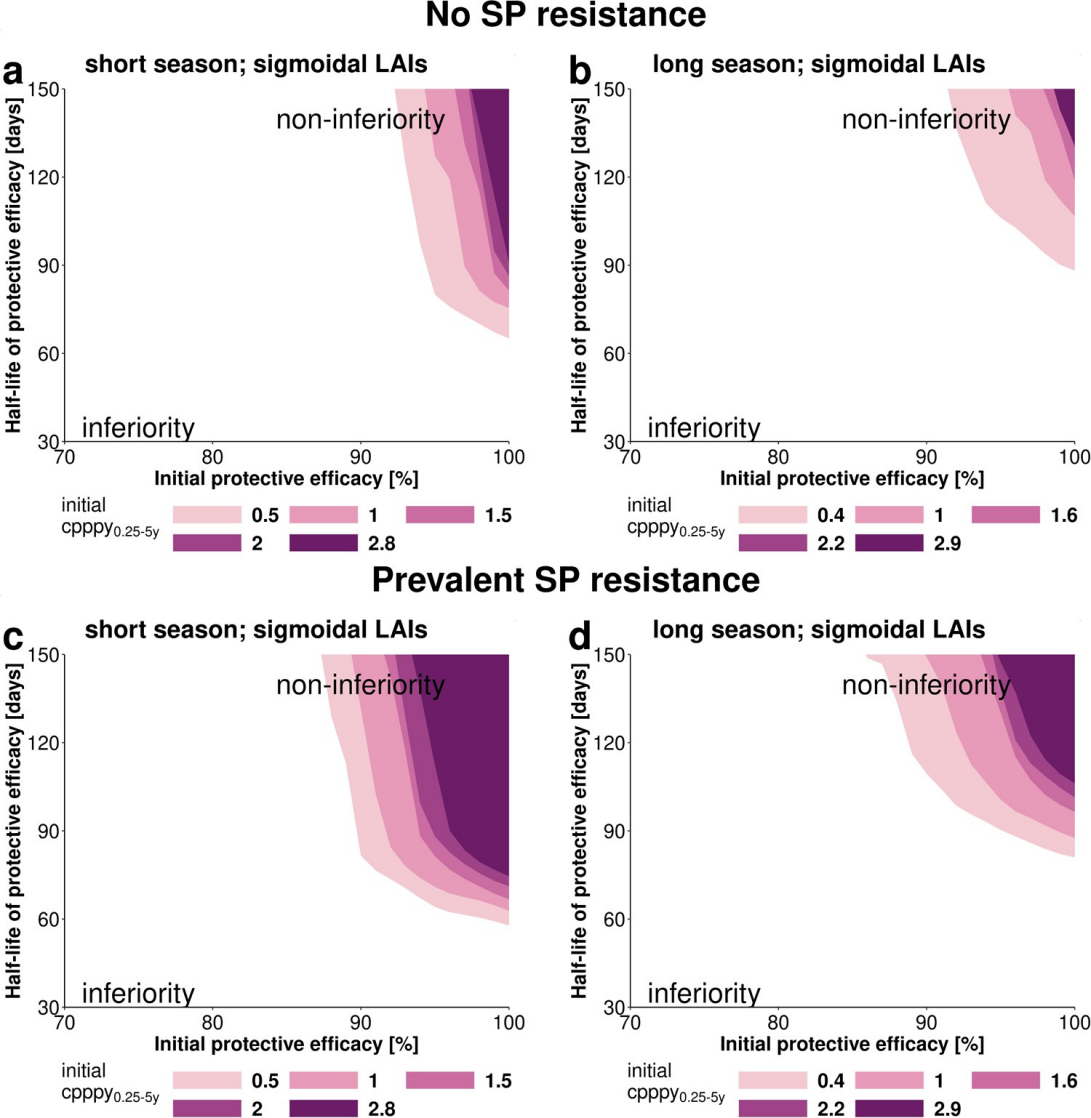
Parameter space under which *sigmoidal LAIs* achieve non-inferiority compared with SMC-SP+AQ in the *clinical trial stage*. We investigated the ability of *sigmoidal LAIs* to establish non-inferiority in clinical trials with an optimal deployment coverage (100%) in settings with a short (a, c) and long (b, d) malaria season and over varying initial malaria incidence (initial cases per person per year_0.25-5y_). The minimum required LAI characteristics are defined as those parameter combinations that achieve non-inferior for different baseline incidence settings. (a, b) SMC–SP+AQ has an initial protective efficacy of 100% and a half-life of 32 days parameterised from previous clinical trial data^1^. The influence of prevalent SP-resistance (c, d) was analyzed by decreasing the protective efficacy half-life of SP from 32 to 20 days (see S1 Text). The coloured area defines the limits of the parameter space where non-inferiority could be established through comparison of the difference hazard ratio δ between Kaplan-Meier survival estimates (see S1 Text). The white area describes the parameter space where LAIs are inferior. *Sigmoidal LAIs* can achieve non-inferiority compared with SMC for lower durations of protection in a shorter malaria transmission season. *Exponential LAIs* and *biphasic LAIs* could not establish non-inferiority to SMC–SP+AQ.

In the *clinical trial stage*, we discretized the space of the initial protective efficacy and protective efficacy half-life of LAIs within the range of plausible values (Table 1), yielding a two-dimensional grid of parameter value combinations. At each grid point, we used the GP emulators to estimate the upper limit of the confidence interval of the hazard ratio between the survival estimates in the SMC and LAI arm and the non-inferiority margin and check if non-inferiority could be established for the given combination of parameter values (cf. Non-inferiority Analysis in S1 Text). The contours of the resulting non-inferiority surface yielded the thresholds of the minimal initial protective efficacy and protective efficacy half-life needed to establish non-inferiority across different transmission settings (Fig 2).

In the *implementation stage*, we additionally determined the minimum required LAI coverage and half-life at which non-inferiority to SMC-SP+AQ could be established. At each grid point, we conducted a constrained optimisation, translating the non-inferiority condition into an inequality constraint by requiring the difference between the upper limit of the confidence interval and the non-inferiority margin to be positive. The optimisation was conducted using an augmented Lagrange method (*gosolnp*, R-package *Rsolnp*, Version 1.16) with a minimum of 3 starting values and 200 simulations. To determine the benefits of reallocation of resources from reduced visits within a season towards increasing deployment coverage, we compared the number of cases per person per year in a simulation with SMC implemented at a given coverage to the number of cases per person per year with LAI at a coverage that was 20% higher than the optimal coverage.

## 3. Results

### Decay shape and duration of protective efficacy influence LAI impact

**Fig 3:**
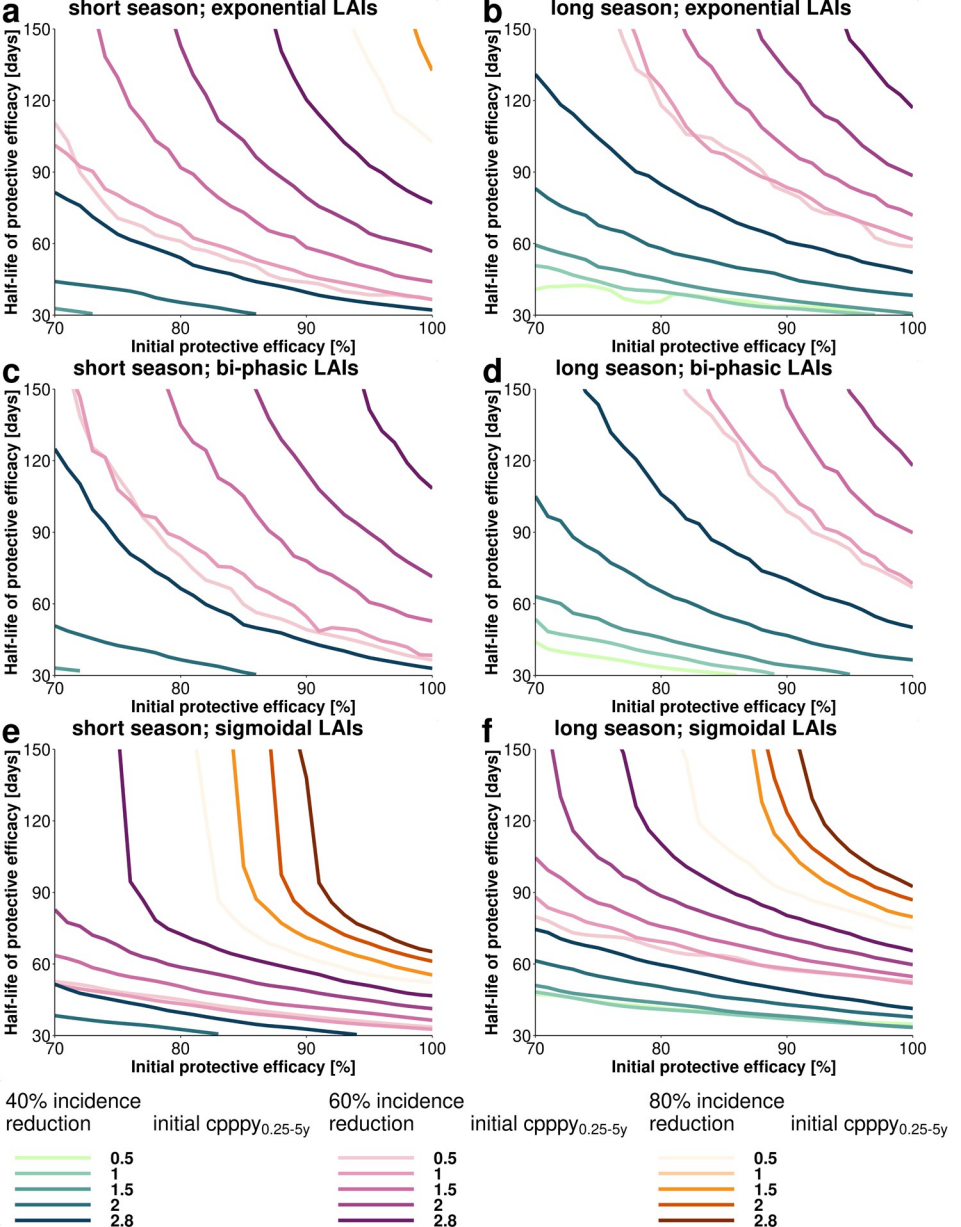
Achieving targeted malaria incidence reduction depends on the decay shape of the LAIs protective efficacy. Estimated relationships between initial protective efficacy and efficacy half-life for different incidence reduction criteria (40%, 60% and 80%, line style and color) and clinical incidence settings (increasing color intensity indicates an initial clinical incidence measured in cases per person per year in the target age group of 0.5, 1, 1.5, 2, and 2.8). Each line shows the minimum required LAI characteristics to reach the desired health goal at a 100% LAI deployment coverage at *clinical trial stage*, with all parameter combinations below a line failing to meet those requirements. The panels show the parameter space of attainable incidence reductions within the specified constrained ranges of initial protective efficacy and half-life for *exponential LAIs* (a, b), *biphasic LAIs* (c, d) and *sigmoidal LAIs* (e, f) in settings with a short (Senegal-like a, c, e) or long (Mali-like b, d, f) malaria season. The incidence reduction was calculated by comparing the incidence over one transmission season after application of the LAI compared with the previous transmission season. The incidence reductions were obtained by predicting the cases per person per year_0.25-5y_ via our emulator approach (See S1 Text) in a fine grid defined over the parameter space (increments of 0.1 h for half-life and 0.01% for initial protective efficacy) and calculating the incidence reduction by comparison to the initial clinical incidence measured in cases per person per year_0.25-5y_ in the respective transmission intensity setting.

We assessed the obtained malaria incidence reduction in the simulated scenarios of the *clinical trial stage* at high deployment coverage (100%) and found that the decay shape of LAI protective efficacy and protection half-life play an important role in achieving a targeted incidence reduction (Fig 3) for each simulated clinical trial scenario. Additionally, we identified the parameter space in which a certain incidence reduction cannot be achieved following LAI deployment under different setting assumptions (below each curve in Fig 3).

In settings with a more extended transmission season (Fig 3B, 3D and 3F), a longer LAI half-life is required to reach the same predicted impact compared with the shorter season settings (Fig 3B, 3C and 3E). A steeper initial decrease of initial protective efficacy (Fig 1A and Fig C in S1 Text) led to lower estimated incidence reduction, with *biphasic LAIs* exhibiting the lowest predicted impact (Fig 3C and 3D), followed by *exponential LAIs* (Fig 3A and 3B). The predicted impact of *sigmoidal LAIs* was largely determined by their half-life compared with initial protective efficacy: an initial decrease in protective efficacy would require only a marginal increase of the half-life needed to reach the desired incidence reduction for half-lives greater than 90 days (Fig 3E and 3F). In contrast to *exponential* (Fig 3A and 3B) and *biphasic LAIs* (Fig 3C and 3D), where the force of infection increased the required minimum half-life of protective efficacy, the required minimum half-life for *sigmoidal LAIs* was not markedly increased by the force of transmission compared with the exponential and biphasic decays (Fig 3E and 3F). A longer malaria transmission season (Fig 3B, 3D and 3F) was predicted to increase the half-life requirements to reach a desired incidence reduction for all LAIs. In these longer transmission season settings, a predicted incidence reduction of over 80% was not possible for *exponential* and *biphasic* LAIs.

Fig 3 shows the exemplary extraction of minimum essential efficacy properties for a LAI with a given half-life. For example, if the half-life of protective efficacy of a LAI was assumed to be 150 days, we predicted that an initial protective efficacy of 88%, 96%, and 76% was required for *exponential*, *biphasic* and *sigmoidal LAIs*, respectively (Fig 3A, 3C and 3E) to reach a clinical incidence reduction of 60% (short malaria transmission season, initial cases per person per year_0.25-5y_ of 2.8).

### Establishing non-inferiority of LAIs to SMC-SP+AQ in clinical trials is difficult

In the SMC-SP+AQ arm of the simulated *clinical trial stages*, we found a predicted mean achievable incidence reduction of 71% to 90% in Mali and Senegal-like seasonal settings (Table C in S1 Text). Our non-inferiority analysis (Fig 1B panel 3) demonstrated that the predicted establishment of non-inferiority of *sigmoidal LAIs* to SMC-SP+AQ under the assumption of 100% initial deployment coverage could only be met with LAI efficacy over 90% in both seasonal settings and half-life over 62 days in Senegal-like (short season) and 88 days in Mali-like (long season) seasonal transmission patterns (Fig 2). In agreement with the analysis of attainable incidence reduction (Fig 3), the predicted establishment of non-inferiority was more feasible in settings with a shorter transmission season and lower initial malaria incidence (Fig 2A). For settings with a lower initial incidence (between 0.5 and 1 initial cases per person per year_0.25-5y_), the parameter space where non-inferiority could be established varied more than in higher initial incidence settings. If resistance against SP was prevalent, which we modelled as a shorter duration of protection through a decrease in protective efficacy half-life to 20 days (from 32 days), *sigmoidal LAIs* were predicted to be non-inferior in a wider range of tool property combinations (Fig 2C and 2D). Nevertheless, non-inferiority could not be established in any setting for any parameterization of *exponential* and *biphasic LAIs* under clinical trial coverage assumptions. We conclude that the efficacy decay profiles of LAIs play an important role for reaching the defined incidence reduction goals and establishing non-inferiority to SMC-SP+AQ.

### The influence of protective efficacy half-life changes over the parameter space

Moving from clinical field trials towards *implementation stages*, where LAIs are administered as a replacement for SMC-SP+AQ (Fig 1B panel 2), we analyzed the influence of underlying LAI efficacy properties and deployment coverage on resulting intervention impact and non-inferiority to SMC-SP+AQ.

**Fig 4:**
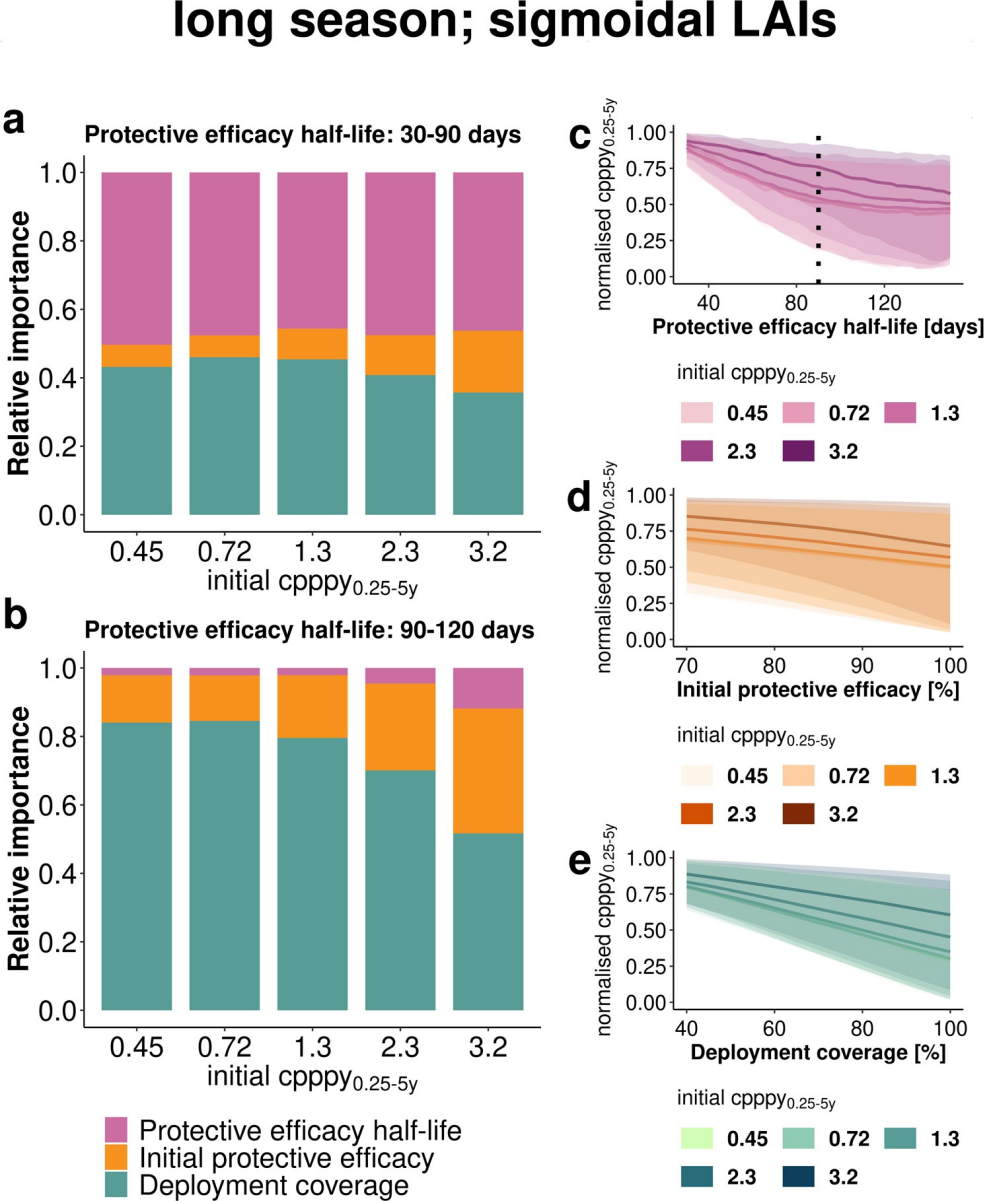
Estimated importance of LAI properties and operational factors on the level of clinical incidence reduction. Results are shown for the *implementation stage* for *sigmoidal LAIs* in a setting with low access to care and long malaria transmission season. (a, b) Sobol sensitivity analysis estimates the relative importance of LAI coverage, initial protective efficacy and half-life of protective efficacy to the variance of the emulator through decomposition of variance over the entire evaluated parameter space (coverage 40–100%, initial protective efficacy 70–100% and half-life (a) 30–90 days and (b) 90–150 days). Changes in clinical incidence measured as cases per person per year_0.25-5y_ with increasing tool properties or deployment coverage across the parameter space are shown for (c) half-life (30–150 days), (d) initial protective efficacy (70–100%) and (e) coverage (40–100%). The lines represent the mean and the 95% confidence interval (shaded area) capture the distribution of incidence reduction across all sampled values. The dotted line in panel c indicates the split of half-life range for sensitivity analysis in panels a and b. Increasing color intensity represents increasing initial cases per person per year_0.25-5y_. Further results for different decay shapes and length of transmission season are shown in the Figs D and E in S1 Text.

Our sensitivity analysis via decomposition of variance (Fig 4) indicates that the influence of half-life of protective efficacy depends on the length of the transmission season in *implementation stages*. An increase in initial efficacy (Fig 4D) and deployment coverage (Fig 4E) resulted in a linear increase in predicted impact. In contrast, the influence of the protective efficacy half-life changed over the parameter space (Fig 4A–4C), with a much steeper influence for changes in lower half-life ranges. We found a change in the main source of variance of the impact of *sigmoidal LAIs* with increasing protective efficacy half-life (shown in Fig 4A and 4D for a half-life threshold of 90 days). For instance, if the LAI half-life was less than 90 days (Fig 4A), the protective efficacy half-life and deployment coverage shared almost equal proportions of attributable variance, accounting for 38% to 52% of variance while the initial protective efficacy contributed between 7% and 19%, depending on transmission intensity. However, for half-lives greater than 90 days (Fig 4B) the importance of deployment coverage increased from 54% to 85% and importance of the initial protective efficacy from 14% to 38% (in these results we assumed LAIs of less than 70% initial protective efficacy are unlikely to be developed). In contrast, the relative importance of the protective efficacy half-life decreased to around 2% to 12%. In settings with a longer malaria transmission season (Fig F panels A, D, and G in S1 Text), a sharper initial decrease in clinical incidence was predicted for a larger range of protective efficacy half-life than for shorter transmission seasons (Fig E panels A, D, and G in S1 Text). Overall, this demonstrates the potential to explore how the impact determinants and their importance change based on efficacy duration cutoffs compared with the length of transmission season or alternative deployment.

### Trade-offs between enhancing duration of protection and implementation coverage

These results illustrate the importance of setting-specific trade-offs between enhancing tool properties and improving implementation coverage (Table 2). For example, increasing the half-life of a *sigmoidal LAI* with an initial efficacy of 90% from 49 to 63 days reduced the predicted required LAI coverage to establish non-inferiority to SMC-SP+AQ in implementation (60% coverage) by 20% (from 100 to 80%) in a setting with an initial clinical incidence of 1.4 cases per person per year_0.25-5y_. Furthermore, in settings with relatively high levels of initial clinical malaria incidence and corresponding high transmission intensity, namely cases per person per year_0.25-5y_>2.4 and EIR>150, a change in dynamics to establish non-inferiority was observed. In these settings, we predicted LAIs will likely fail to sufficiently protect the targeted population from clinical malaria even at very high deployment coverage (Fig D in S1 Text). Therefore, we were unable to assess the required half-life of protective efficacy of LAI for high transmission settings.

**Table 2:** Illustration of the trade-offs between LAI protective efficacy half-life, initial protective efficacy and coverage in *implementation stages*.

		*Exponential LAIs* Coverage [%]				*Sigmoidal LAIs* Coverage [%]			
Efficacy [%]	initial cases per person per year_0.25-5y_	40%	60%	80%	100%	40%	60%	80%	100%
70%	0.41	-	-	113	62	-	-	85	59
	0.71	-	-		70	-	-	87	63
	1.4	-	-	-	89	-	-	103	63
	2.4	-	-	-	93	-	-	105	79
	3.3	-	-	-	-	-	-	-	-
80%	0.41	-	-	109	54	-	-	71	52
	0.71	-	-	112	62	-	148	74	55
	1.4	-	-	132	64	-	-	77	57
	2.4	-	-	119	75	-	-	84	62
	3.3	-	-	-	-	-	-	-	-
90%	0.41	-	-	81	48	-	102	61	50
	0.71	-	-	79	52	-	86	64	52
	1.4	-	-	90	56	-	103	63	49
	2.4	-	-	92	62	-	97	72	56
	3.3	-	-	-	-	-	-	-	-
100%	0.41	-	112	62	46	-	78	58	47
	0.71	-	115	69	48	-	78	58	49
	1.4	-	131	71	49	-	78	58	47
	2.4	-	119	72	52	-	80	61	47
	3.3	-	-	-	-	-	-	-	-

The table displays the estimated minimum half-life of LAI protective efficacy (measured in days, estimated values specified in the colored cells, increasing colour intensity illustrates increasing requirements) required to reach non-inferiority in implementation stages of various LAI profiles compared with SMC- SP+AQ deployed at 60% coverage for each of 3 or 4 rounds in a setting with low access to healthcare (E_14_ = 0.1). SMC-SP-AQ protective efficacy specifications are summarized in Table 1. Results are shown for different levels of LAI coverage, decay shapes (exponential or sigmoidal), initial protective efficacy and malaria incidence prior to deployment (initial cases per person per year_0.25-5y_).

For SMC-SP+AQ, we estimated that an additional 13% to 29% in incidence reduction could be achieved by increasing the coverage from 62% to 100%, dependent on initial clinical incidence before implementation (Fig G in S1 Text). However, achieving high levels of SMC coverage at implementation is challenging [6,7], and increasing levels of coverage are associated with increasing costs.

**Fig 5:**
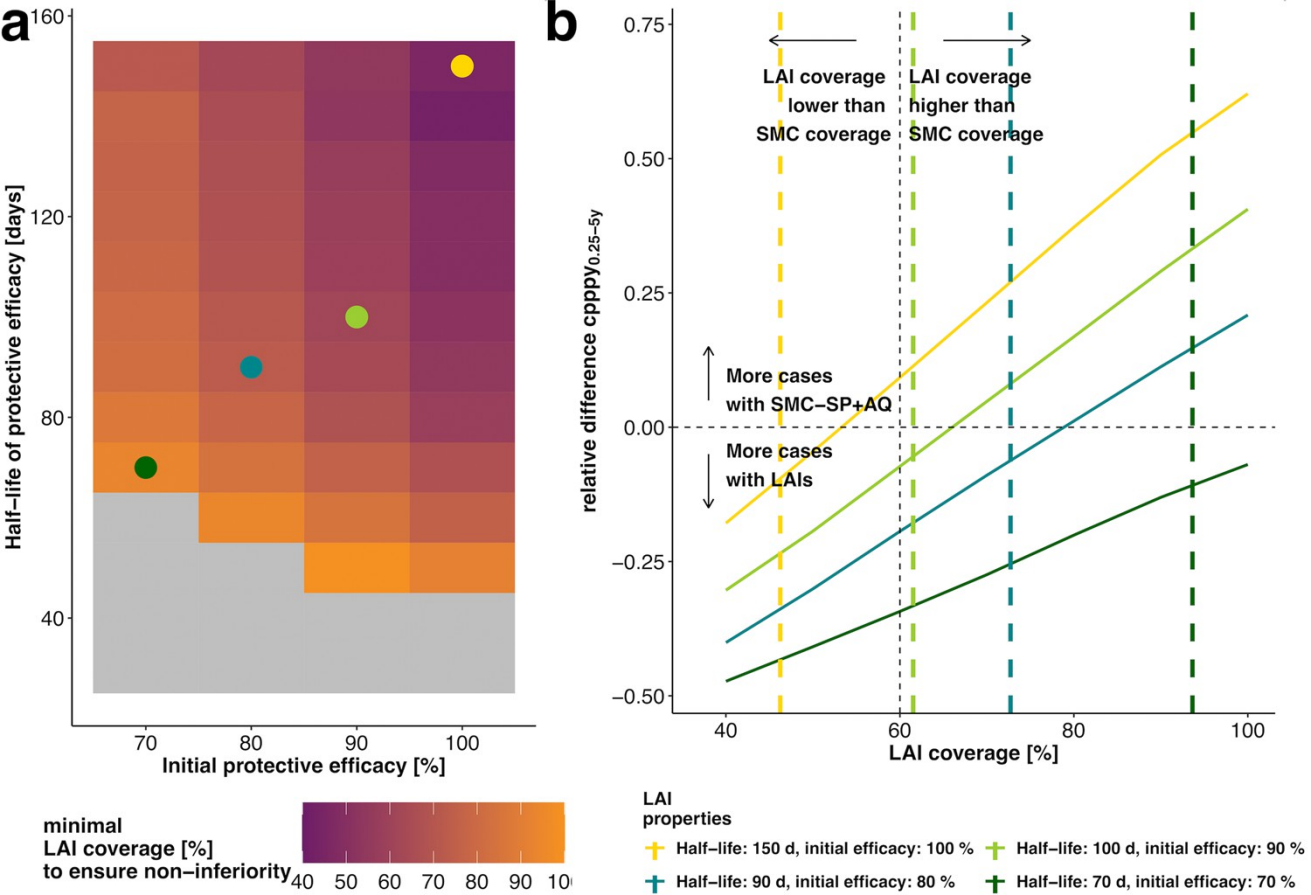
Estimated minimal LAI coverage required during *implementation stages* to achieve non-inferiority in a given setting and predicted gains in cases averted of subsequent *sigmoidal* LAI coverage increments. (a) Heatmap of the estimated minimal coverage (colour) of *sigmoidal LAIs* at which non-inferiority to SMC-SP+AQ (assuming a fixed SMC coverage of 60%) is achieved for different combinations of *sigmoidal LAI* efficacy and half-life. The results are displayed for intervention scenarios with an underlying disease burden of 1.4 cases per person per year_0.25-5y_, long malaria transmission season and low access to treatment (E_14_ = 0.1). In the grey area, non-inferiority of LAIs could not be established for any coverage. The light blue frames capture the tool characteristics where non-inferiority could be reached with a LAI coverage under the reference SMC-SP+AQ coverage of 60%. Further results for additional settings and decay shapes are provided in the S1 Text (Fig H and I in S1 Text). The coloured dots represent four illustrative LAI profiles for which the corresponding predicted relative differences in cases per person per year_0.25-5y_ (Eq 5) are calculated in (b-e) five years after LAI introduction over all LAI coverages as compared with SMC-SP+AQ at 60% coverage (vertical dotted line). The predicted positive increase in relative difference in yearly clinical cases (above the dotted horizontal line) means more clinical cases are averted with LAIs than with SMC-SP+AQ. It thus illustrates the benefit of increasing *sigmoidal LAI*-coverage above the minimal required coverage to achieve non-inferiority (shown by the grey coloured area). Due to the chosen margin of non-inferiority (here 5%, see Material and Methods), LAIs are non-inferior for a slight negative relative difference in cases per person per year_0.25-5y_. In the light-blue area in (b), a LAI coverage lower than the SMC-SP+AQ coverage is sufficient to establish non-inferiority. The corresponding analysis for *exponential LAIs* can be found in Fig J in S1 Text.

As information on costs of LAI (costs of goods and supply chain) are not available as of now, we were unable to include detailed economic analysis in the assessment of LAIs in *implementation stages*. The main cost drivers of SMC-SP+AQ are deployment costs (remuneration of healthcare workers) and cost of goods [51,52], with deployment costs increasing non-linearly with higher coverage. Therefore, we determined the minimal LAI coverage at which non-inferiority to SMC-SP+AQ (assuming initial SMC deployment coverage of 60%) was established, stratified by initial clinical incidence before implementation (Fig 5 and Figs H and I in S1 Text). We found that the parameter space where non-inferiority could be established shrank with increasing baseline malaria incidence (Figs H and I in S1 Text). With regard to seasonality, LAIs were more likely to be non-inferior in shorter malaria transmission settings in the *implementation stage* (Figs G and H in S1 Text). In settings with a high initial clinical incidence (EIR = 150, Table A in S1 Text), LAI coverage could not be optimised to be non-inferior to SMC-SP+AQ at 60% coverage because LAIs were unable to prevent malaria cases even at very high coverage levels (Fig D panel D in S1 Text). We did not explore less seasonal profiles or transmission profiles with reasonable ongoing transmission in the dry season. However, longer duration LAIs might potentially be needed to reach non-inferiority for periods of evaluation extending into the dry season. Overall, the optimisation of LAI coverage in settings with a limited SMC-SP+AQ coverage illustrates the potential of LAI implementation in non-optimal coverage settings.

While non-inferiority could only be established in a small part of the parameter space of *sigmoidal LAIs* in long seasonal transmission settings (Fig 2B), it is possible to increase the potential area of applicability of *sigmoidal LAIs* by optimising their deployment coverage (Fig 5A, initial cases per person per year_0.25-5y_ = 1.4). Deploying a sigmoidal *LAI* at 46% coverage with a half-life of 150 days and initial efficacy of 100% is sufficient to establish non-inferiority over SMC-SP+AQ at 60% coverage (Fig 5B). In contrast, *sigmoidal LAIs* with a half-life of 70 days and initial efficacy of 70% require a deployment coverage of 95% in order to be non-inferior (Fig 5E). For *sigmoidal LAIs* we found that increasing the deployment coverage over the estimated minimum required coverage to establish non-inferiority results in potential gains in terms of clinical incidence reduction compared with SMC-SP+AQ (Fig 5B–5E).

Additionally, we found that even though non-inferiority of *exponential LAIs* to SMC-SP+AQ could not be established in *clinical trial stages*, coverage optimisation in *implementation stages* reveals their applicability. Deploying an *exponential LAI* at 52% coverage with a half-life of 150 days and initial efficacy of 100% was sufficient to establish non-inferiority over SMC-SP+AQ at 60% coverage (Fig J panel B in S1 Text) and a half-life of 100 days and initial protective efficacy of 90% requires a deployment of 78% to establish non-inferiority (Fig J panel C in S1 Text). *Exponential LAIs* with a half-life of 70 days and initial efficacy of 70% were always inferior to SMC-SP+AQ at a coverage of 60% (Fig J panel E in S1 Text).

## 4. Discussion

The effective prevention of clinical malaria in children is crucial to prevent malaria mortality and reduce the overall global malaria burden [1]. Through modelling and simulation, we explored a broad range of LAI characteristics in multiple settings for clinical testing and deployment. This allowed us to understand the likely influence of LAI efficacy properties and operational factors on clinical incidence reduction in children under five years of age when LAI is deployed as a seasonal malaria prevention tool. We found that if the protective efficacy of a new LAI decays immediately after injection, for example an exponential or biphasic-like decay, then the LAI is unlikely to achieve non-inferiority over SMC-SP-AQ in current SMC settings in a clinical trial. This exploration assumed non-inferiority criterion is required for testing, and we only explored LAI half-life of protection in the range of 30 to 150 days. In contrast, when the protective efficacy of LAIs is long-lasting and decays only after some time (i.e. a sigmoidal decay), there is a stronger chance of achieving non-inferiority when the duration of protection is close to the transmission length. Beyond clinical stages, by assessing implementation factors versus LAI properties, we conclude that focusing on enhancing the duration of protective efficacy (half-life) is more likely to result in successful LAIs in a larger range of incidence and transmission settings. If the half-life of protective efficacy of a LAI approaches the length of transmission season, and initial efficacy is sufficiently high (depending on the transmission intensity (Fig 3)), then the development of new LAI should prioritize optimising operational delivery factors to ensure reasonable coverage to be as good as or better in averting clinical cases than current SMC-SP+AQ implementation.

### The estimated impact of tool properties

In general, the duration of the half-life and its shape of decay are the most relevant tool properties for incidence reduction. This means that the development of new LAIs should focus on understanding the likely decay and half-life of protection (this is not the pharmacokinetic properties of a small molecule or the antibody longevity of a mAb). Over the analyzed transmission settings and parameter space, the estimated minimum essential (80%) and ideal (95%) incidence reduction targets for LAIs as defined by Macintyre et al. (2018) [17] are hard to achieve in clinical testing. A desired incidence reduction of >80% in clinical testing could only be achieved for LAIs with a sigmoidal decay shape and half-life over 60 days (Senegal, short season) and 80 days (Mali, long season). Our findings indicate that key to identifying and refining candidates for development of new LAIs is investigating the decay shape of efficacy as early as possible and providing a sufficiently long protection half-life. Before conducting large scale clinical trials, it will therefore be important to ensure the adequate means to establish the decay and duration of protection and the ability to extrapolate to paediatric indications.

We investigated three very different decay profiles of potential LAI protection because this information is currently unknown. While the pharmacokinetic profile of potential LAIs can be evaluated in pharmacokinetic studies, their protective efficacy profile and decay shape are harder to derive. Currently, murine [53] or human challenge [54,55] studies are being used to investigate these parameters. In these studies, subjects receive treatment before inoculation with sporozoites through the bites of infectious mosquitoes or direct venous injection [54,55]. However, parasite growth in the liver, and therefore protective efficacy, cannot be directly quantified. Instead, the time and number of parasites entering the blood stream is used as a crude proxy for protective efficacy. We note, however, that sporozoite or mosquito human challenge studies with pharmacokinetic modelling will provide valuable information to refine the profiles of protection against infection investigated in our study. These data and PKPD models refining protective efficacy decay curves and incorporating them into the population modelling approach with *OpenMalaria* can provide more informed first insights into the potential public health impact of new LAIs.

In summary, our findings suggest that when defining key efficacy characteristics for TPPs for LAIs, there are two important processes that should be carried out: (1) evaluate the feasibility of existing or newly specified efficacy and duration requirements for LAIs [17] by estimating their public health impact using for example, modeling and simulation approaches, and (2) translate the population-level protective efficacy of LAIs on the incidence of clinical cases into individual-level protection efficacy, and vice versa, using reasonable measures of protective efficacy from summations of animal or human studies.

### Clinical non-inferiority trials

Our non-inferiority analysis (Fig 2) highlights that the establishment of non-inferiority in *clinical trial stages* is challenging due to not only the high protective ability of SMC-SP+AQ but also clinical trial designs. This motivates an important discussion on the clinical development of LAIs under the use-case of SMC replacement: the necessity and extent of clinical trial testing or non-inferiority criteria should be assessed. We estimated SMC-SP+AQ in a clinical study results in incidence reductions of 71% to 90% in children under five, aligning to a efficacy of 86% (95% CI 78–91%) in a clinical trial in Senegal [56]. Monthly SMC-SP+AQ administration together with the estimated protective efficacy half-life of 32 days offers a high degree of protection and the blood-stage clearing effect of AQ eliminates remnant malaria infections, making optimally employed SMC a very powerful tool to prevent clinical malaria. This means it is difficult for most LAIs to achieve non-inferiority in a clinical trial with limited SP resistance when the LAI is only deployed once per season in combination with a blood stage clearing drug. Thus, at first glance, our non-inferiority results may offer a distorted picture of the ability of LAIs to compete with current SMC, as clinical non-inferiority trials do not reflect the reality of SMC implementation. Operational constraints result in reduced coverage over the number of rounds [5,6] and adherence [11], thus hindering SMC-SP+AQ from reaching its full potential. As SMC-SP+AQ effectiveness reduces with operational challenges, this presents a niche for LAIs. In 2018, among children under 5 years of age living in SMC-eligible areas in the 12 countries in the Sahel subregion that scaled-up SMC, only 62% (19 of 31 million) were administered SMC-SP+AQ [1], meaning that additional incidence reduction could be achieved by increasing coverage at each round.

### Tool and coverage optimisation in implementation stages

Replacing SMC-SP+AQ with LAIs will likely prove beneficial in reducing deployment costs due to fewer deployment rounds. In the absence of information on costs of LAIs (costs of goods and supply chain), we are unable to adequately assess economic considerations and thus our results assume that coverage is the main driver of implementation cost. If LAIs are assumed to have a longer clearance half-life and therefore higher protective efficacy for longer than SP+AQ, resources are freed up through decreased deployment rounds within a season. These resources could be reallocated to increase the overall coverage in a single round of LAI in the target population, including populations in remote places. Additionally, the overall adherence to the blood-stage clearing co-administration of antimalarials could be increased, thereby reducing the probability of emergence of resistance. However, if transmission intensity is very high, we found that the optimisation of protective efficacy half-life and deployment coverage is insufficient to adequately protect the targeted population. Instead, it might be necessary to expand the deployment of LAIs to multiple administration rounds within a transmission season.

The optimisation of deployment coverage of LAIs to reach non-inferiority to SMC-SP+AQ where optimal coverage cannot be met exposes an additional use case for LAIs. If external circumstances, such as the current COVID-19 pandemic, prevent the regular implementation of SMC and bed-nets campaigns, millions of children will experience an increased risk of malaria [57], LAIs may alleviate this burden. Our analysis can aid the identification of minimal LAI coverages necessary to achieve given population impacts and prevent a resurgence in malaria cases.

### Framework and areas of application

Our modelling and simulation analysis provides important insights into the likely impact of new malaria tools that are currently under clinical development. Our modelling framework divides the unknown parameter space of realistic properties of new tools into setting-specific attainable incidence reduction by translating the decay of individual protection against infection of a new tool into estimates of population impact on clinical incidence. While in clinical trials often only one (high) deployment coverage and a limited number of trial-arms can be investigated, a simulation-based approach can explore the trade-off between operational constraints and tool properties to narrow down beneficial implementation settings and use cases without the need for expensive field studies. Not only does this approach offer the opportunity to assess the potential population impact of new tools currently under development, but it also provides a methodology to assess the potential clinical trial outcomes. It assists the evaluation of clinical trial scenarios that might be considered over several different malaria transmission and health-system settings to supporting thinking on appropriate population impact endpoints that are suitable to inform decision making. This is particularly true for existing interventions with high efficacy for which the establishment of non-inferiority in clinical non -inferiority trials is problematic, due to the required large sample sizes [58]. Here, our approach offers first insights into the outcomes of such trials and the additional possibility to develop clinical trial analysis tools.

Furthermore, beyond the current scope of our study, as more information on likely costs of LAIs become available and further certainty in implementation and cold-chain needed, this work can serve as a basis for cost-effectiveness or economic analysis.

As with all modelling studies there are limitations to our analysis. In this study, despite exploring a large range of characteristics on tool properties, deployment, and transmission settings, our results are constrained by the investigated parameter-space. First, we only investigated the impact of one administration round of LAIs with an antimalarial treatment and assumed that the time-point of administration would coincide with the first SMC application round. However, depending on the LAI profile, the time of deployment may need to be optimised. Second, the implementation of protective efficacy of LAIs is solely assumption-driven, as clinical data is not yet available. With additional information on likely achievable protective efficacy half-life, initial protective efficacy, and efficacy decay shapes, the preliminary LAI profiles can be further defined and re-evaluated as LAIs are developed. The re-definition of plausible parameter ranges will also impact the results of the sensitivity analysis and trade-offs, and potentially shift the recommended focus of development efforts. Additionally, target mediated drug disposition might change the pharmacokinetic profile of mAbs dependent on the transmission intensity and parasite growth within the human host and therefore also its efficacy profile [59]. Third, the focus of this analysis was the investigation of the effect of the anti-infective LAIs. Our results are subject to change if LAIs are co-administered with different blood-stage clearing drugs (different efficacies and/or potential properties e.g. transmission blocking) or are deployed with other interventions such as insecticide-treated bed-nets. And lastly, we explored SMC or LAI replacement in only children under 5 years of age and in settings similar to where SMC is currently deployed such as Mali and Senegal. Further analysis could be undertaken to assess LAI as seasonal prevention in children under 10 years of age, however we expect conclusions to be similar in regards tool properties and coverage requirements. Our results also only hold for assessing LAI as replacing SMC; we did not explore use cases of deploying LAI in perennial or other settings in which SMC is not yet deployed. Alternative clinical metrics would need to be explored as LAI in these use-cases are not a replacement tools, rather new tools and non-inferiority trials are not relevant. Although this study focuses on the use of LAIs in seasonal malaria transmission settings, our findings regarding the importance of protective efficacy half-life do provide first insights for potential use of a LAIs in perennial malaria transmission settings. The protective efficacy half-life of a LAI will most likely dictate the number of applications to children during the yearly seasonal transmission to ensure effective clinical case reduction.

### Current stage of development of mABs and duration of protection

Potential candidate LAIs include mABs and small-molecule drugs, and to date most known mAbs for use in malaria (largely by-products from research into whole sporozoite vaccines [60]) have been shown to prevent blood-stage infection in *in vitro* and/or *in vivo* murine malaria infection experiments [20,21]. As the natural clearance half-life of antibodies ranges between 2 and 21 days [61], strategies to increase the half-life of mAbs have been introduced via modifications to the tail (Fc) region of antibodies that interacts with the receptors on the surface of cells. Fc-modified mAbs have exhibited extended half-lives ranging from, 100 days [62] and 80 to 112 days [63] in healthy human adults. Our results suggest that these extended half-lives, if functional malaria protection is maintained, are crucial to establish non-inferiority to standard SMC. A second stream of LAI development is focused around small-molecule drugs such as atovaquone [18] and P218 [19]. Although these compounds show promising liver-stage activity, the estimated clearance half-life of enhanced formulation atovaquone of 32 days in humans [18] and 8.9–19.6 hours of P218 in first-in-human trials [64] are again likely insufficient in the use cases explored in our study and emphasize the need for longer-lasting formulations to be useful as SMC replacements when deployed only once per season. Further use may be possible for multiple applications within a season.

## 5. Conclusion

Here, we provided the first quantitative evaluation of the TPPs of future LAIs for malaria as a seasonal prevention tool in children. Simulation analysis of LAIs in real-life implementation settings revealed that the ability of LAIs to prevent clinical cases in children is strongly dependent on the length of the malaria transmission season and transmission intensity. We also found it is important to focus on improving the protective efficacy duration (half-life) of LAIs in development, as the speed of protective efficacy decay is a key driver of overall impact (a crucial consideration for TPP development) or the chance to meet non-inferiority criteria compared with SMC-SP+AQ. However, if a reasonable duration is possible (longer half-life and sigmoidal decay that supports protection close to the length of transmission season) then development should focus on increasing deployment coverage to optimise the LAIs chance of higher impact. This provides evidence for the potential trade-offs between tool properties and operational constraints as LAIs are developed and deployed. In general, our findings support the need for a thorough and combined investigation of tool properties and use cases in the future development of LAIs. This combined effort includes earlier modelling alongside clinical studies to provide evidence of translation of impact at population levels before late stage clinical studies and optimise the success of new malaria tools. Our research here provides an initial foundation to support dialogue between stakeholders, scientists, and clinicians at each clinical development stage of novel anti-infective LAI’s to reduce clinical malaria incidence. LAIs have the potential to be a game changer in protecting vulnerable populations from malaria. Our analysis serves as a stepping stone for the refinement of TPPs for LAIs, thereby assisting the target-oriented use-case of development and implementation of new LAIs.

## Supporting information

S1 Text Fig A: Prevalence—incidence relationship in the simulated Open Malaria settings. Fig B: Modelled malaria transmission pattern and simulated prevalence defining seasonality settings. Fig C: Simulated protective efficacy decay shapes of long acting injectables (LAI) and seasonal malaria chemoprevention (SMC) over one transmission season. Fig D: Exemplary illustration of incidence and survival estimates of sigmoidal LAIs and SMC-SP+AQ in implementation stages over one implementation year. Fig E: Estimated impact of LAI properties and operational factors on the level of clinical incidence reduction. Fig F: Estimated impact of LAI properties and operational factors on the level of clinical incidence reduction. Fig G: Incidence reduction achieved through implementation of SMC-SP+AQ over varying deployment coverage. Fig H: Estimated minimal LAI coverage required during implementation stages to achieve non-inferiority in a given setting. Fig I: Estimated minimal LAI coverage required during implementation stages to achieve non-inferiority in a given setting. Fig J: Estimated minimal LAI coverage required during implementation stages to achieve non-inferiority in a given setting and predicted gains in cases averted of subsequent exponential LAI coverage increments. Fig K: True over predicted RSS of the GP. Fig L: Decay of protective efficacy of SMC-SP+AQ over time. Fig M: Cumulative hazard of malaria in children who received SMC with SP-AQ (blue) compared to controls (black). Fig N: Prevalence of malaria in children who received SMC with SP-AQ (blue) compared to controls (black) over the trial. Table A: Simulated prevalence–incidence settings. Table B: Emulator performance for the investigated outcomes. Table C: Incidence reduction achieved through implementation of SMC-SP+AQ in a clinical trial setting. Table D: Inputs into OpenMalaria (OM) to check adequate parameterization of SMC-SP-AQ. Table E: Results of the GP optimization. Table F: Comparison of trial results of Zongo et al., 2015 3 and model outputs using the model specification in Table D with the best parameter set.
(PDF)

## Abbreviations

SMC: Seasonal malaria chemoprevention
LAI: Long-acting injectable
TPP: Target product profile
mAB: Monoclonal antibody
SP+AQ: Sulfadoxine-Pyrimethamine + Amodiaquine
GP: Gaussian process
