# Supplementary material for: Model-informed target product profiles of long-acting-injectables for use as seasonal malaria prevention
Source: PLOS Glob Public Health. 2022 Mar 14;2(3):e0000211. doi: 10.1371/journal.pgph.0000211 (PMC10021282; doi:10.1371/journal.pgph.0000211)
Supplement: S1 Text — Fig A: Prevalence—incidence relationship in the simulated Open Malaria settings. Fig B: Modelled malaria transmission pattern and simulated prevalence defining seasonality settings. Fig C: Simulated protective efficacy decay shapes of long acting injectables (LAI) and seasonal malaria chemoprevention (SMC) over one transmission season. Fig D: Exemplary illustration of incidence and survival estimates of sigmoidal LAIs and SMC-SP+AQ in implementation stages over one implementation year. Fig E: Estimated impact of LAI properties and operational factors on the level of clinical incidence reduction. Fig F: Estimated impact of LAI properties and operational factors on the level of clinical incidence reduction. Fig G: Incidence reduction achieved through implementation of SMC-SP+AQ over varying deployment coverage. Fig H: Estimated minimal LAI coverage required during implementation stages to achieve non-inferiority in a given setting. Fig I: Estimated minimal LAI coverage required during implementation stages to achieve non-inferiority in a given setting. Fig J: Estimated minimal LAI coverage required during implementation stages to achieve non-inferiority in a given setting and predicted gains in cases averted of subsequent exponential LAI coverage increments. Fig K: True over predicted RSS of the GP. Fig L: Decay of protective efficacy of SMC-SP+AQ over time. Fig M: Cumulative hazard of malaria in children who received SMC with SP-AQ (blue) compared to controls (black). Fig N: Prevalence of malaria in children who received SMC with SP-AQ (blue) compared to controls (black) over the trial. Table A: Simulated prevalence–incidence settings. Table B: Emulator performance for the investigated outcomes. Table C: Incidence reduction achieved through implementation of SMC-SP+AQ in a clinical trial setting. Table D: Inputs into OpenMalaria (OM) to check adequate parameterization of SMC-SP-AQ. Table E: Results of the GP optimization. Table F: Comparison of trial results of Zongo [file pgph.0000211.s001.pdf]

Supplementary material for

Model informed target product profiles of long-acting-injectables for use as seasonal malaria prevention

Authors:

Lydia Burgert[1, 2], Theresa Reiker[1, 2], Monica Golumbeanu[1,2], Jörg J. Möhrle[1, 2, 3], Melissa A. Penny*[1, 2]

[1] Swiss Tropical and Public Health Institute, Basel, Switzerland

[2] University of Basel, Basel, Switzerland

[3] Medicines for Malaria Venture, Geneva, Switzerland

*Corresponding author: melissa.penny@unibas.ch

Table of Contents

1. General OpenMalaria specification and intervention set-up 3

2. Non-inferiority analysis 6

3. Emulator performance 7

4. Additional analysis results in the clinical trial setting 7

5. Additional analysis results in the implementation setting 8

6. Parameterization of SMC-SP+AQ to clinical trial data 14

1. General OpenMalaria specification and intervention set-up

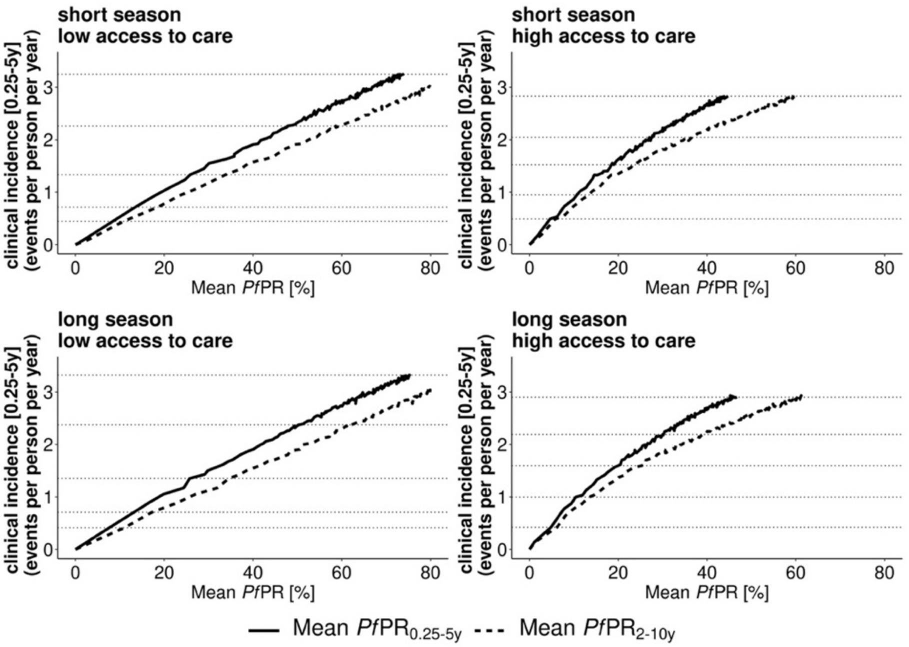

Fig A: Prevalence - incidence relationship in the simulated Open Malaria settings. The relationship between prevalence and incidence is displayed in the two simulated seasonal settings (Senegal/short season and Mali/long season) and health system access settings (low and high access) (Table 1) in absence of any interventions. The clinical incidence defined as the events per person per year in the target age-group (0.25-5 years of age) is shown for the corresponding mean prevalence over one year in the intervention age group (*Pf*PR$_{0.25\text{-}5y}$) and in children between 2-10 years (*Pf*PR$_{2\text{-}10y}$). The dotted horizontal lines mark the incidence settings, simulated for all downstream analyses and can be found in Table A.

Table A: Simulated prevalence – incidence settings. The force of transmission was defined by the entomological inoculation rate (EIR: mean number of infectious bites per person per year (pppy)). Across different transmission settings defined by access to healthcare (HC), EIR levels, and transmission seasonality, corresponding simulated annual mean malaria prevalence (*Pf*PR) and clinical incidence per person per year in the intervention age group are displayed for different age groups: 2 to 10 year olds (*Pf*PR$_{2\text{-}10y}$) and 0.25 to 5 years old (*Pf*PR$_{0.25\text{-}5y}$ and cases per person per year$_{0.25\text{-}5y}$). The adjustment of EIR between HC access settings ensures similar levels of case incidence simulated across transmission season settings.

		**Short transmission season**			**Long transmission season**		
**HC Access**	**EIR** [infectious bites pppy]	***Pf*PR $_{2\text{-}10y}$** [%]	***Pf*PR $_{0.25\text{-}5y}$** [%]	**Cases per person per year$_{0.25\text{-}5y}$**	***Pf*PR $_{2\text{-}10y}$** [%]	***Pf*PR $_{0.25\text{-}5y}$** [%]	**Cases per person per year$_{0.25\text{-}5y}$**
**high**	5	6	5	0.49	6	5	0.42
	9	13	11	0.95	13	10	1
	20	23	19	1.52	25	20	1.6
	47	37	28	2.04	39	30	2.2
	150	60	45	2.83	62	46	2.9
**low**	3	11	8	0.45	11	8	0.41
	4	19	13	0.72	17	13	0.71
	8	34	26	1.3	35	26	1.4
	28	59	48	2.3	62	50	2.4
	150	86	73	3.2	87	75	3.3

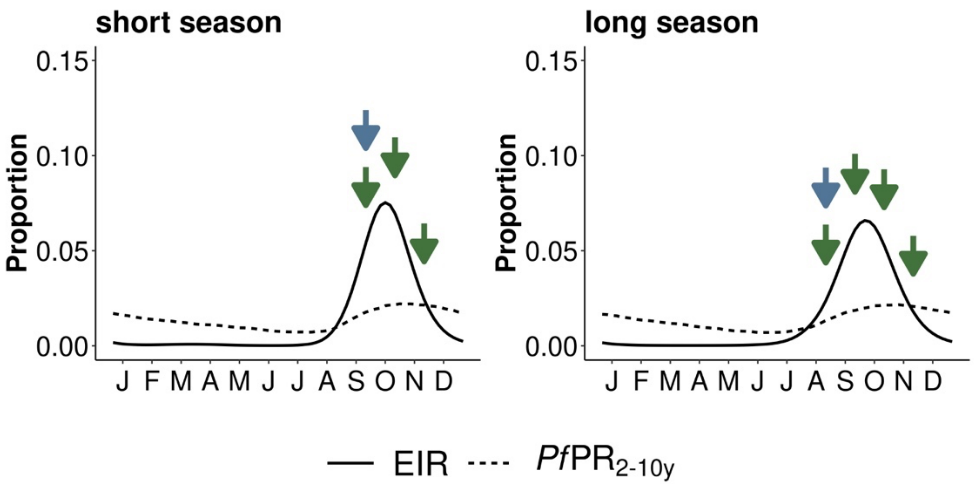

Fig B: Modelled malaria transmission pattern and simulated prevalence defining seasonality settings. The seasonality in malaria transmission is defined through the proportional EIR (solid line) in relation to the yearly EIR (input into the simulator). Here shown for a yearly EIR of 100 infectious bites per person per year. The dotted line represents the resulting scaled yearly prevalence profile. The arrows illustrate the administration of the preventative interventions of SMC-SP+AQ (green) and LAI (blue) in the two seasonal settings. Following WHO recommendations[1], SMC-SP+AQ was implemented monthly with the first dose administered before the peak and the second dose with the peak in malaria transmission.

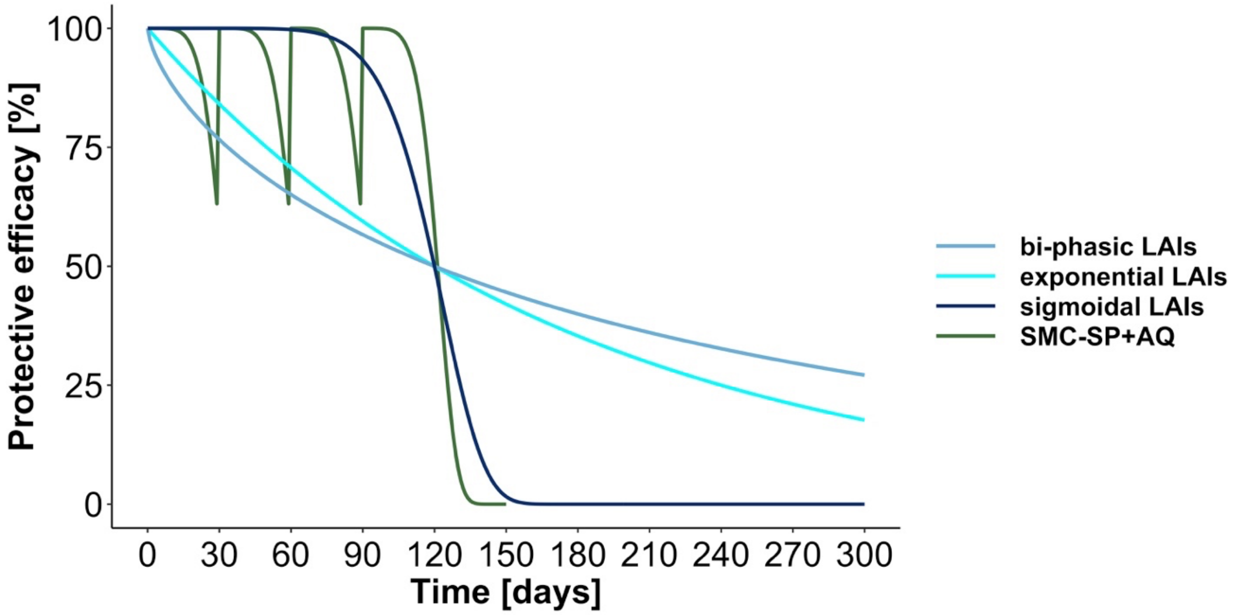

Fig C: Simulated protective efficacy decay shapes of long acting injectables (LAI) and seasonal malaria chemoprevention (SMC) over one transmission season. The LAI decay shapes were chosen such that they represent the three possible development streams of mAb (sigmoid decay), drug based (exponential decay), or vaccine like (biphasic) LAIs (blue solid line). Here, we illustrated a LAI protective efficacy half-life of 120 days and initial protective efficacy of 100 %. SMC – SP+AQ was parameterised as specified below and administered up to four times over the transmission season (green solid line). The parameterisation of the interventions is further specified in tablet.

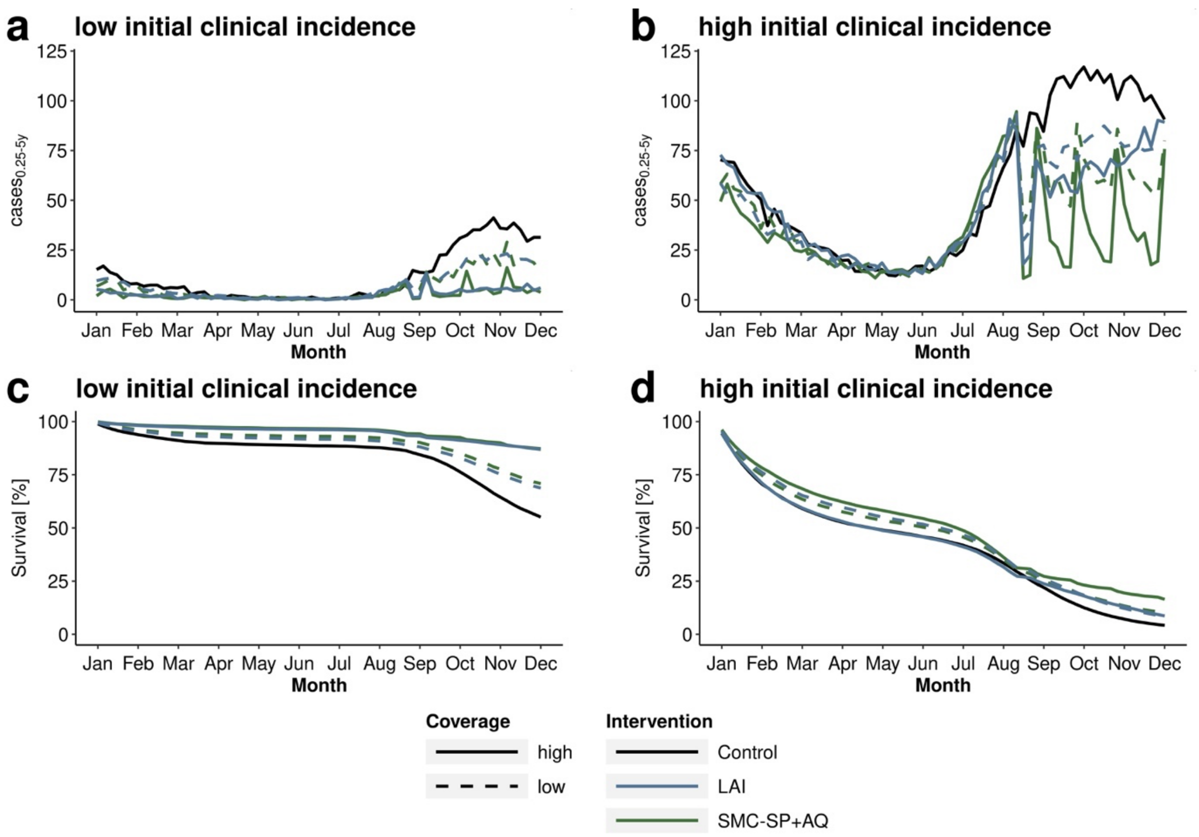

Fig D: Exemplary illustration of incidence and survival estimates of *sigmoidal LAIs* and SMC-SP+AQ in *implementation stages* over one implementation year. Plots are shown in **(a, c)** low initial clinical incidence settings (initial cases per person per $year_{0.25\text{-}5y}$ = 0.71) and **(b, d)** high initial clinical incidence settings (initial cases per person per $year_{0.25\text{-}5y}$ = 3.3) in long malaria transmission settings with low access to healthcare (E_{14}=0.1). The *sigmoidal LAIs* exhibit an initial protective efficacy over 95 % and protective efficacy half-life of 112 days. SMC-SP+AQ and LAIs were implemented at high deployment coverage (>90%, solid lines) and low deployment coverage (<50%, dashed lines). In low initial incidence settings **(a, c)** SMC-SP+AQ (green) is administered four times per season (Aug-Nov) with clinical incidence notably decreasing after each administration **(a, b)** compared to control settings with no implemented interventions (black). In contrast, the *sigmoidal LAIs* are only administered once at the beginning of the transmission season. With progressing season, the protective efficacy decays, resulting in an increase of clinical cases **(a, b)** and decrease in survival estimates **(c, d)** after LAI administration. In low clinical incidence settings, *sigmoidal LAIs* and SMC-SP+AQ are comparably effective at both coverage levels **(c)**. In contrast, the survival estimates in in high initial clinical incidence settings **(d)** reveal that *sigmoidal LAIs* at both coverage levels and SMC-SP+AQ at low coverage levels are all equally unable to prevent malaria cases. Shown here are the mean predictions over 5 stochastic *OpenMalaria* simulations.

2. Non-inferiority analysis

Survival analysis was performed using a Kaplan-Meier approach as specified in [2], with the K-M estimate $\hat{S}(t)$ derived via number of new clinical cases c_i at each time step t_i

$$\hat{\boldsymbol{S}}(\boldsymbol{t}) = \prod_{i:t_i \leq t} \mathbf{1} - \frac{c_i}{N_{int}}, \qquad \textbf{Equ. S1}$$

with the standard error $\widehat{SE}$ calculated using the Greenwood formula

$$\widehat{\boldsymbol{SE}}(\boldsymbol{t}) = \left[\hat{\boldsymbol{S}}(\boldsymbol{t})\right] \sqrt{\prod_{i:t_i \leq t} \frac{c_i}{N_{int}(N_{int}-c_i)}}. \qquad \textbf{Eq. S2}$$

Non-inferiority analysis was conducted based on the survival statistics as described in [2]. The survival estimate (hazard ratio) for the standard of care SMC $\hat{S}_{SMC}$ (t) considering the desired margin of non-inferiority Δ defines the upper limit for non-inferiority γ :

$$\boldsymbol{\gamma} = \frac{\ln(\hat{S}_{SMC}-\Delta)}{\ln(\hat{S}_{SMC})} \qquad \textbf{Eq. S3}$$

The difference δ between the survival estimates of the standard of care SMC $\hat{S}_{SMC}$ (t) and new treatment (LAI) $\hat{S}_{LAI}$, is calculated on the log-log- scale and is equivalent to the logarithm of the ratio of cumulative hazards in the two groups

$$\delta = \ln\left(-\ln\left(\hat{S}_{SMC}\right)\right) - \ln\left(-\ln\left(\hat{S}_{LAI}\right)\right) = \ln\frac{\widehat{H}_{SMC}(t)}{\widehat{H}_{LAI}(t)}$$

Eq. S4

and its variance is calculated as follows:

$$Var(\delta) = \left\{\frac{1}{\ln(\hat{S}_{SMC})}\right\}^2 \frac{1}{\hat{S}_{SMC}{}^2} Var\left(\hat{S}_{SMC}\right) + \left\{\frac{1}{\ln(\hat{S}_{LAI})}\right\}^2 \frac{1}{\hat{S}_{LAI}{}^2} Var\left(\hat{S}_{LAI}\right) \qquad \textbf{Eq. S5}$$

The confidence interval of the hazard ratio δ is given by $exp^{\delta\ [95\%\ CI:\ \delta \pm 1.96 \times se(\delta)]}$. Non-inferiority is established if the upper limit of the derived 95% confidence interval, CI_{high}, of the hazard ratios δ between SMC and LAI lies below the upper limit for non-inferiority γ.

3. Emulator performance

Table B: Emulator performance for the investigated outcomes. The emulator performance was assessed by calculating the Pearson correlation coefficient (PCC) and the mean absolute error (MAE) between true and predicted values on a 20% holdout set for each investigated setting.

Outcome	PCC (median [min, max])	MAE (median [min, max])
**upper limit for non-inferiority γ**	0.9924 [0.8072, 0.9972]	0.0015 [0.0002, 0.0280]
**upper limit of the hazard ratio**	0.9945 [0.9472, 0.9978]	0.0229 [0.0094, 0.0791]
**Clinical incidence SMC-SP+AQ, (cases per person per $year_{0.25\text{-}5y,\ SMC}$)**	0.9964 [0.9787, 0.9986]	0.0142 [0.0060, 0.0428]
**Clinical incidence LAIs, (cases per person per $year_{0.25\text{-}5y,\ LAI}$)**	0.9947 [0.9562, 0.9985]	0.0136 [0.0055, 0.0488]

4. Additional analysis results in the clinical trial setting

Table C: Incidence reduction achieved through implementation of SMC-SP+AQ in a clinical trial setting. The mean incidence reduction was calculated in the clinical trial setting over three months in the short season setting and four months in the long season setting. Resistance to SMC-SP+AQ is implemented as a reduction in protective efficacy half-life from 32 to 20 days (half-life of 32 days parameterised from previous clinical trial data[3]).

		**Short malaria transmission season**	**Long malaria transmission season**
	**EIR** [infectious bites pppy]	**inc_{red}** [%]	**inc_{red}** [%]
**No resistance to SMC-SP+AQ**	5	90	87
	9	90	87
	20	90	88
	47	89	87
	150	88	86
**Resistance to SMC-SP+AQ**	3	79	76
	4	76	75
	8	76	72
	28	73	72
	150	73	71

5. Additional analysis results in the implementation setting

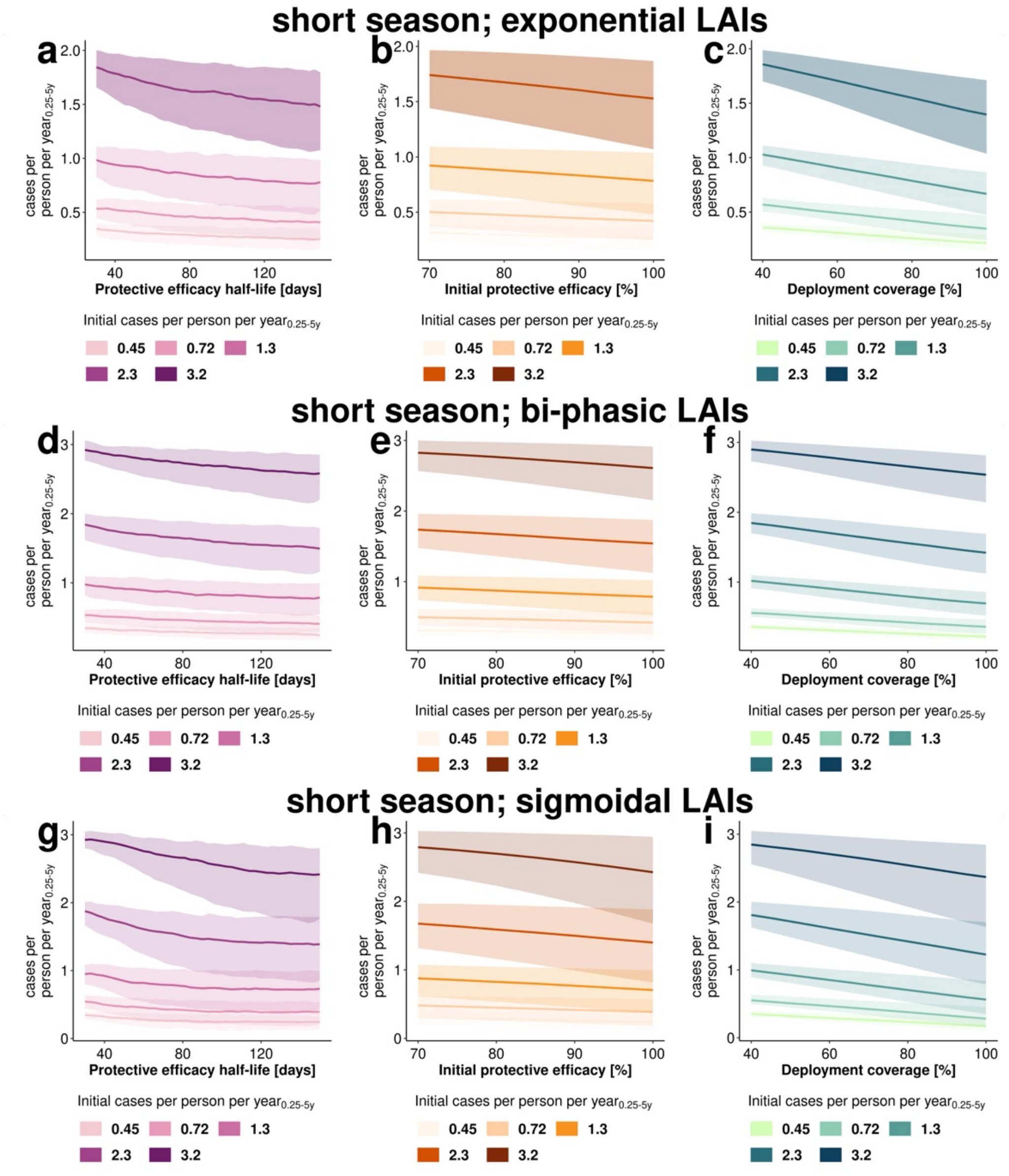

Fig E: Estimated impact of LAI properties and operational factors on the level of clinical incidence reduction. Results are shown for the implementation stage in a setting with low access to care (E_{14}=0.1) and short (Senegal-like) malaria transmission season, for **(a-c)** *exponential LAIs*, **(d-f)** *biphasic LAIs*, and **(g-i)** *sigmoidal LAIs*. Changes in clinical incidence measured as cases per person per $\text{year}_{0.25\text{-}5y}$ with increasing tool properties or deployment coverage across the parameter space are shown for for **(a, d, g)** half-life (30-150 d) , **(b, e, h)** initial efficacy (80-100%) and **(c, f, i)** coverage (40 -100 %). The lines represent the mean and the 95%- confidence bands (shaded area) capture the distribution of incidence reduction across all sampled values. Increasing color intensity represents increasing initial cases per person per $\text{year}_{0.25\text{-}5y}$). These results hold true for high access to healthcare settings. The conversion of initial cases per person per $\text{year}_{0.25\text{-}5y}$ to prevalence can be found in Table A.

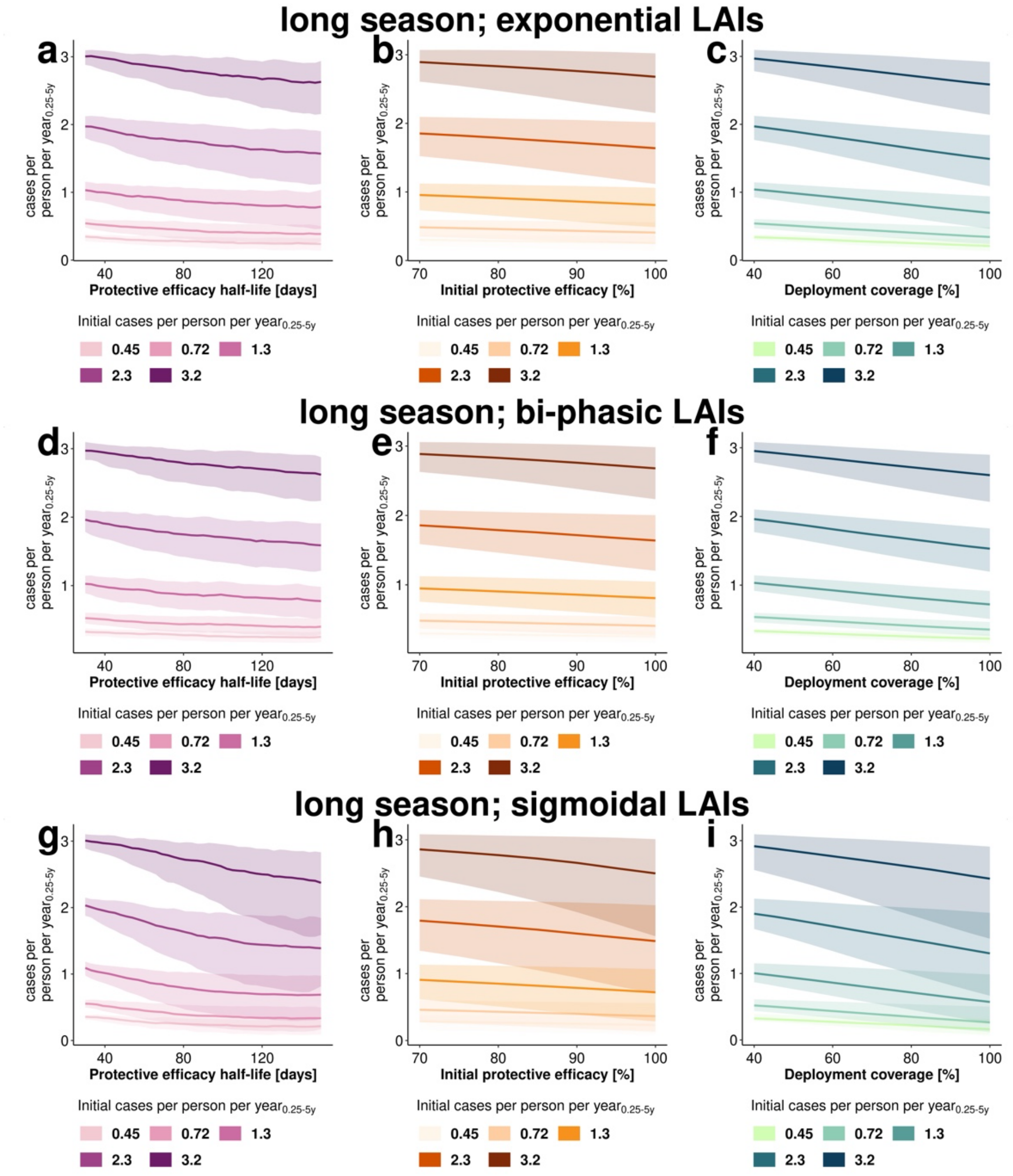

Fig F: Estimated impact of LAI properties and operational factors on the level of clinical incidence reduction. Results are shown for the implementation stage in a setting with low access to care (E_{14}=0.1) and long (Mali-like) malaria transmission season, for **(a-c)** *exponential LAIs*, **(d-f)** *biphasic LAIs*, and **(g-i)** *sigmoidal LAIs*. Changes in clinical incidence measured as cases per person per $year_{0.25\text{-}5y}$ with increasing tool properties or deployment coverage across the parameter space are shown for **(a, d, g)** half-life (30-150 d) , **(b, e, h)** initial efficacy (80-100%) and **(c, f, i)** coverage (40 -100 %). The lines represent the mean and the 95%- confidence bands (shaded area) capture the distribution of incidence reduction across all sampled values. Increasing color intensity represents increasing initial clinical incidence (cases per person per $year_{0.25\text{-}5y}$). These results hold true for high access to healthcare settings. The conversion of initial cases per person per $year_{0.25\text{-}5y}$ to prevalence can be found in Table A.

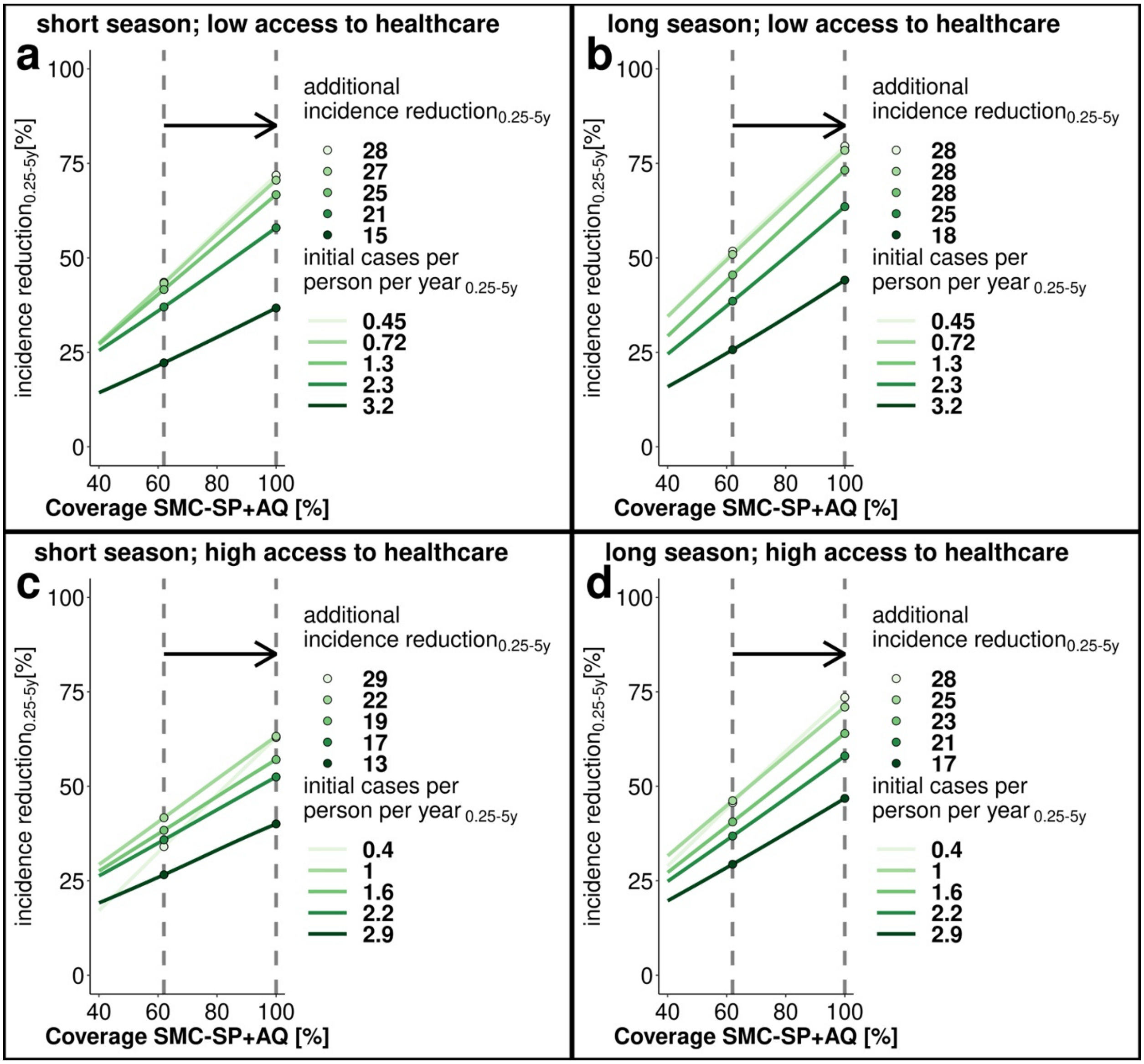

Fig G: Incidence reduction achieved through implementation of SMC-SP+AQ over varying deployment coverage. The results are shown for low **(a, b)** and high **(c, d)** access to healthcare and short **(a, c)** and long **(b, d)** malaria seasons. The colors indicate different initial clinical incidence before introduction of SMC-SP+AQ (initial cases per person per year$_{0.25\text{-}5y}$). The incidence reduction$_{0.25\text{-}5y}$ was calculated in the implementation scenario after one year of implementation. The grey lines indicate the additional incidence reduction $_{0.25\text{-}5y}$) achieved by increasing SMC-SP+AQ coverage from 62% to 100 %.

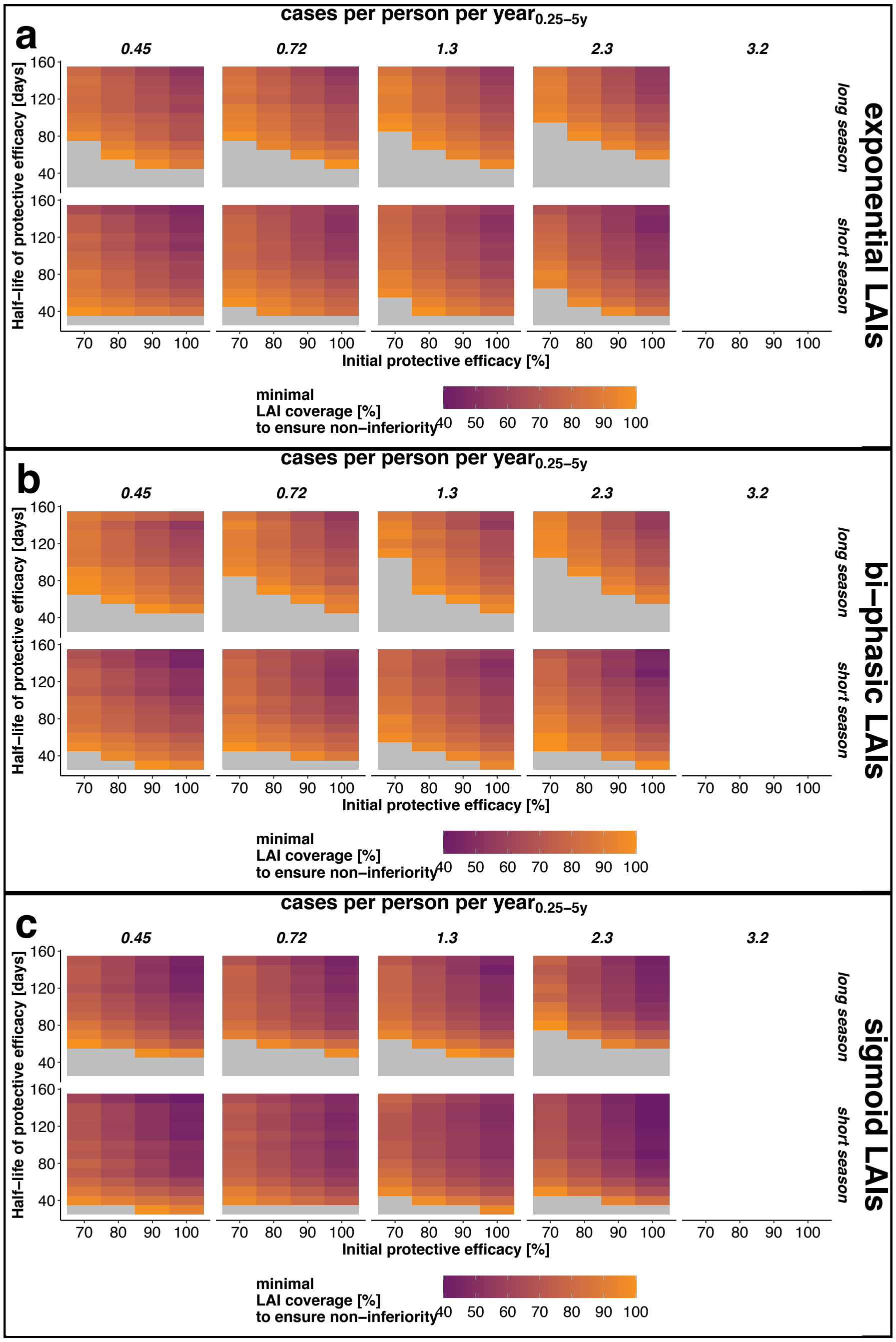

Fig H: Estimated minimal LAI coverage required during *implementation stages* to achieve non-inferiority in a given setting. Heatmap of the estimated minimal coverage (colour) of LAIs at which non-inferiority to SMC-SP+AQ (assuming a fixed SMC coverage of 60% in each of the 3 or 4 rounds) is achieved for different combinations of protective efficacy decay, initial protective efficacy and protective efficacy half-life. The results are displayed for intervention scenarios with a low access to care (E_{14}=0.1) in the two seasonal settings. In the grey area, non-inferiority could not be established for any combination of tool properties. The LAI coverage could not be optimized for high transmission settings (initial cases per person per year$_{0.25\text{-}5y}$=3.2) because they fail to sufficiently protect the targeted population from clinical malaria even at full deployment coverage. Therefore, optimisation of the LAI deployment coverage could not be conducted (Fig D).

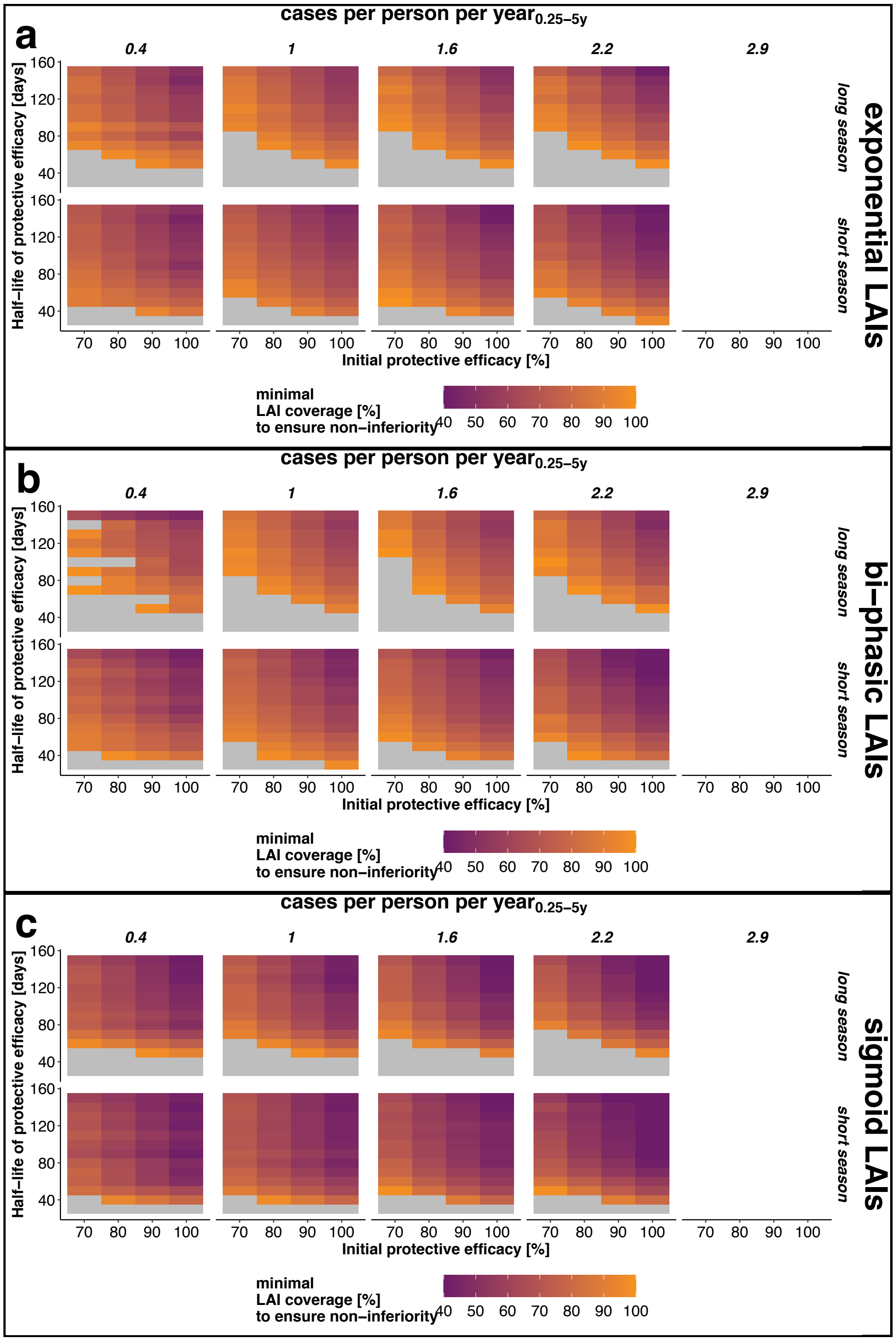

Fig I: Estimated minimal LAI coverage required during *implementation stages* to achieve non-inferiority in a given setting. Heatmap of the estimated minimal coverage (colour) of LAIs at which non-inferiority to SMC-SP+AQ (assuming a fixed SMC coverage of 60% in each of the 3 or 4 rounds) is achieved for different combinations of protective efficacy decay, initial protective efficacy and protective efficacy half-life. The results are displayed for intervention scenarios with a high access to care (E_{14}=0.5) in the two seasonal settings. In the grey area, non-inferiority could not be established for any combination of tool properties. The LAI coverage could not be optimized for high transmission settings (initial cases per person per year$_{0.25\text{-}5y}$=2.9) because they fail to sufficiently protect the targeted population from clinical malaria even at full deployment coverage. Therefore, optimisation of the LAI deployment coverage could not be conducted (Fig D).

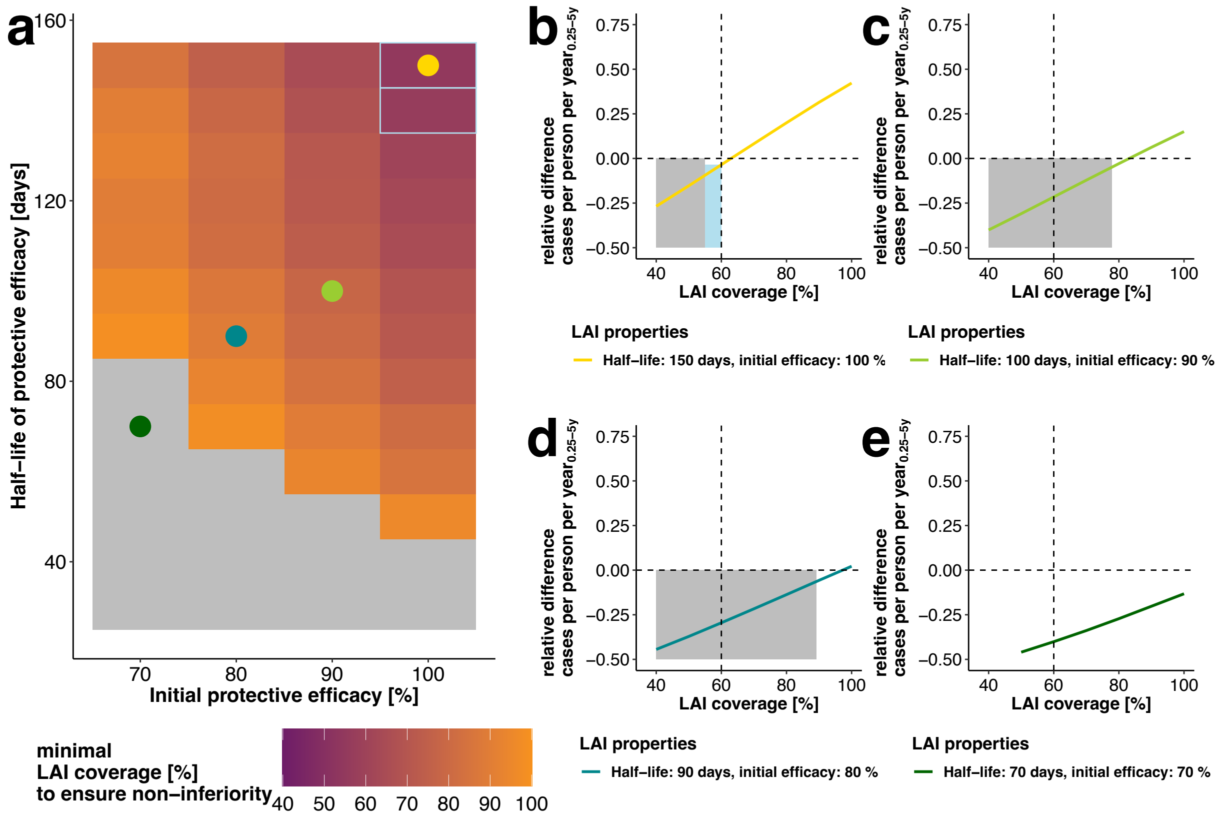

Fig J: Estimated minimal LAI coverage required during *implementation stages* to achieve non-inferiority in a given setting and predicted gains in cases averted of subsequent *exponential* LAI coverage increments. (**(a)** Heatmap of the estimated minimal coverage (colour) of *sigmoidal LAIs* at which non-inferiority to SMC-SP+AQ (assuming a fixed SMC coverage of 60%) is achieved for different combinations of *exponential LAI* efficacy and half-life. The results are displayed for intervention scenarios with an underlying disease burden of 1.4 cases per person per year$_{0.25\text{-}5y}$, long malaria transmission season and low access to treatment (E_{14}=0.1). In the grey area, non-inferiority could not be established for any coverage. For the tool characteristics within the light-blue frames, non-inferiority could be reached with a LAI coverage under the reference SMC-SP+AQ coverage of 60%. The coloured dots represent four illustrative LAI profiles of **(b)**150 days half-life and 100% initial efficacy (yellow), **(c)** 100 days half-life and 90% initial efficacy (light green), **(d)** 90 days half-life and 80% initial efficacy (blue), and **(e)** 70 days half-life and 70% initial efficacy (dark green, e). **(b-e)**. Corresponding predicted relative differences in cases per person per year$_{0.25\text{-}5y}$ (Eq. 5) are calculated for the illustrative LAIs (coloured dots) in (a) in the last implementation year (5 years after LAI introduction) over all LAI coverages as compared to SMC-SP+AQ at 60% coverage (vertical dotted line). The predicted positive increase in relative difference in yearly clinical cases (above the dotted horizontal line) means more clinical cases are averted with LAIs than with SMC-SP+AQ. It thus illustrates the benefit of increasing *exponential LAI*-coverage above the minimal required coverage to achieve non-inferiority (shown by the grey coloured area). In the light-blue area in (b), a LAI coverage lower than the SMC-SP+AQ coverage is sufficient to establish non-inferiority.

6. Parameterization of SMC-SP+AQ to clinical trial data

Seasonal malaria chemoprotection (SMC) was implemented as sulfadoxine–pyrimethamine+amodiquine (SP-AQ) treatment and calibrated to a randomized non-inferiority trial of dihydroartemisinin-piperaquine with SP-AQ, conducted between August 2009 and January 2010 in rural western Burkina Faso Zongo et al.(2015)[3]. Decay of protective efficacy of SPAQ in the field over time was extracted from Fig3 in Zongo et al.(2015)[3] and used to parameterized the decay functions as specified in *OpenMalaria* using a least squares approach combined with a Gaussian-Process optimization. The trial described in Zongo et al.(2015)[1] was rebuilt in OM settings listed in Table D.

Table D: Inputs into OpenMalaria (OM) to check adequate parameterization of SMC-SP-AQ

	Parameter	Value	Ref.
**Vector specification**	Mosquito species [% of total abundance]	*An. funestus*: 42% indoor, 31% outdoor<br>*An. gambiae*: 14% indoor, 13% outdoor	4
	Total EIR	154	4
	Seasonality in malaria transmission [monthly EIR]	*An. funestus*: Aug Sep Oct Nov<br>23 16 0 3.1<br>*An. gambiae*: Aug Sep Oct Nov<br>40 50 16 6.4	4
**Health system**	Access to treatment	2003 2006<br>Effective coverage E_{14} [%] 50 50<br>OM 5-day probability E_5 [%] 24.12 24.12	5, 6
	Malaria treatment	First line: Arthemeter-Lumefantrine<br>Treatment failure/Severe malaria: Quinine	5
**Vector Interventions**	Coverage of ITNs	2006-2008: 14%	5
	ITN coverage during trial	27%	3
**Seasonal malaria chemoprevention**	Timing of interventions	SMC: 15 Aug, 15.Sep, & 15 Oct 2009<br>Follow-up: 1 Sep, 1 Oct & 1 Nov 2009	3
	Protective efficacy of SP+AQ	$E(t) = E_0 \, e^{-\left(\frac{t}{L}\right)^k \log(2)}$<br>Parameter ranges for LHS sampling:<br>Half-life *L*: [20, 60] days<br>Shape parameter *k*: [1, 10]<br>Initial efficacy E_0: [0.7, 1]	3

We used Latin-Hypercube sampling (LHS) to generate 5000 samples of the decay function parameters for protective efficacy (L, k, E_0) within the parameter bounds given in Table D. The trial was simulated with these parameters and five seeds per parameter-set for the intervention and control cohort. The protective efficacy E of SMC - SP+AQ in the simulated trial compared to the simulated controls (without intervention) was extracted by comparing cases per person (cpp) between the intervention group (cpp_{int}) and control group (cpp_{cont}) over the trial period as follows:

$$\boldsymbol{E} = 1 - \frac{\boldsymbol{cpp_{int}}}{\boldsymbol{cpp_{cont}}} \qquad \textbf{Equ. S6}$$

The residual sum of squares (RSS) between the protective efficacy given in Zongo et al.(2015)[3] and protective efficacy from OM simulations was extracted and a Gaussian process (GP) regression was trained to predict the RSS between trial results and OM simulation with the parameters of the protective efficacy decay.

The true over predicted RSS of the hold-out of 1000 data points is shown in Fig K.

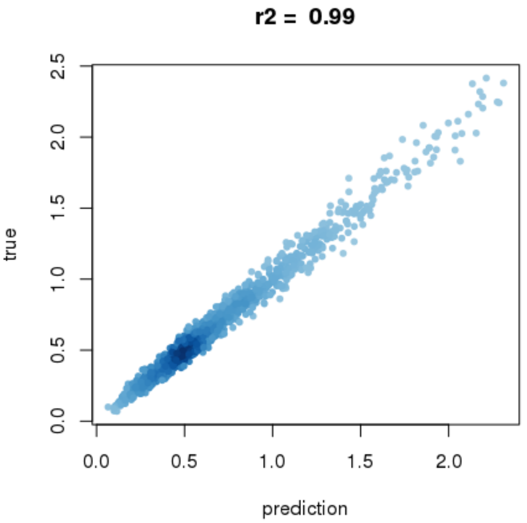

Fig K: True over predicted RSS of the GP.

The GP was then optimized via non-linear optimization using the augmented Lagrange method (function gosolnp, R-package Rsolnp). We optimized the mean predicted error +/- one or two standard deviation of the residual sum of squares (RSS) between the protective efficacy in Zongo et al.(2015)[3] and the OM simulation output. The parameters of the decay function returned by the optimization process were re-simulated with OM and the RSS extracted (*RSS OM*). The OM simulation resulting in the least RSS was then selected for further analysis.

Table E: Results of the GP optimization. The results of the optimization *RSS optim* is compared with the *predicted (pred.) RSS* by the GP and the RSS extracted from OM simulations (median of 5 seeds) conducted with the respective parameters (*RSS OM*). The solver was restarted 20 times and the number of random parameters generated for every restart was set to n.sim=500.

	**kdecay**	**Half-life**	**Initial efficacy**	**RSS optim**	**RSS pred**	**RSS OM**
**Min. RSS before optimisation**	6.35	32.5	0.97	-	0.0842	0.0601
**mean**	5.40	31.3	1.0	0.0538	0.0538	**0.0556**
**Mean +sd**	5.29	31.5	1.0	0.0882	0.0542	0.0583
**Mean - sd**	5.58	31.0	1.0	0.0186	0.0545	0.0538
**Mean + 2*sd**	5.28	31.5	1.0	0.0895	0.0541	0.0556
**Mean – 2*sd**	5.62	31.1	1.0	0.0172	0.0544	0.0739

The OM implementation of the best parameter set for the intervention cohort is able to well capture the protective efficacy described in [3]. The protective efficacy (Fig L), cumulative hazard (Fig M) and prevalence over time (Fig N) provide more insight into the trial results and comparison with OM outputs.

Table F: Comparison of trial results of Zongo et al., 2015 [3] and model outputs using the model specification in Table D with the best parameter set. The compared time-points correspond to the time-points compared in [3]. The difference in prevalence of parasitemia in December 2009 in the untreated cohort are caused by ongoing but decreasing transmission in December (see Fig C) and unclear definition of data time-point in [3].

	Zongo et al., 2015	OM [min, max]
Prevalence of parasitemia [%] in August 2009, intervention cohort	45	45 [44, 48]
Prevalence of parasitemia [%] in September 2009, untreated cohort	61	60 [57, 65]
Cumulative hazard for fever with any parasitemia intervention cohort	0.22	0.14 [0.13, 0.15]
Cumulative hazard for fever with any parasitemia intervention cohort	0.92	1.01 [0.93, 1.12]
Prevalence of parasitemia [%] in November 2009, intervention cohort	12	19 [18, 20]
Prevalence of parasitemia [%] in December 2009, untreated cohort	36	46 [39, 48]

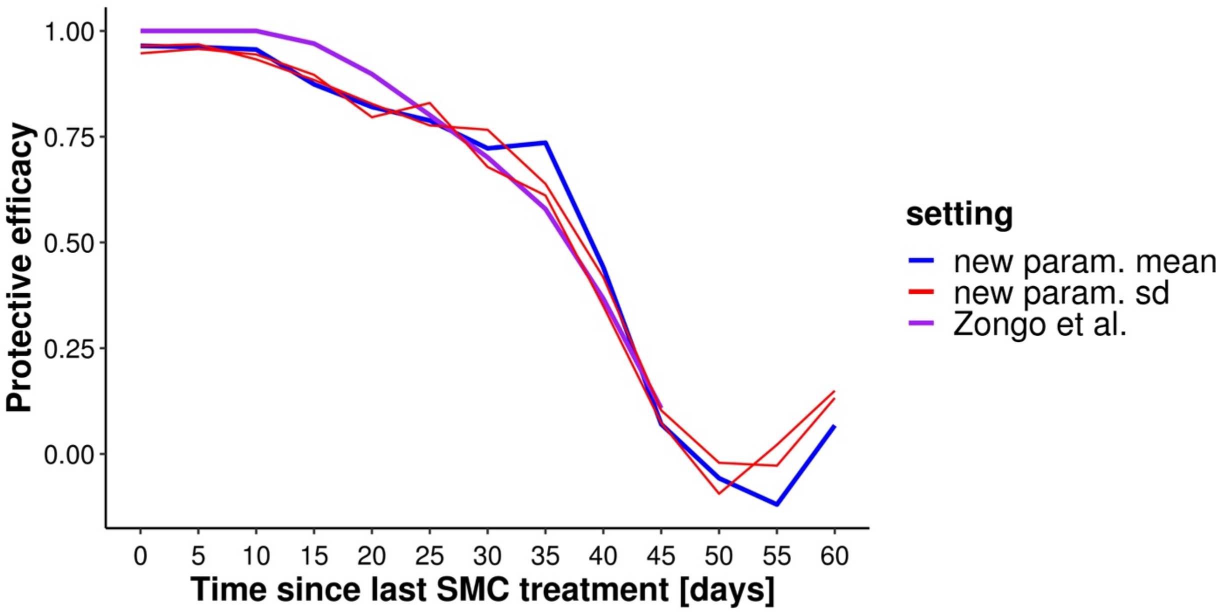

Fig L: Decay of protective efficacy of SMC-SP+AQ over time. Population protective efficacy over time in the trial setting. The purple line represents the protective efficacy as extracted from Zongo et al.(2015) [3]. The new parameterization after optimization and uncertainty are shown in blue and red.

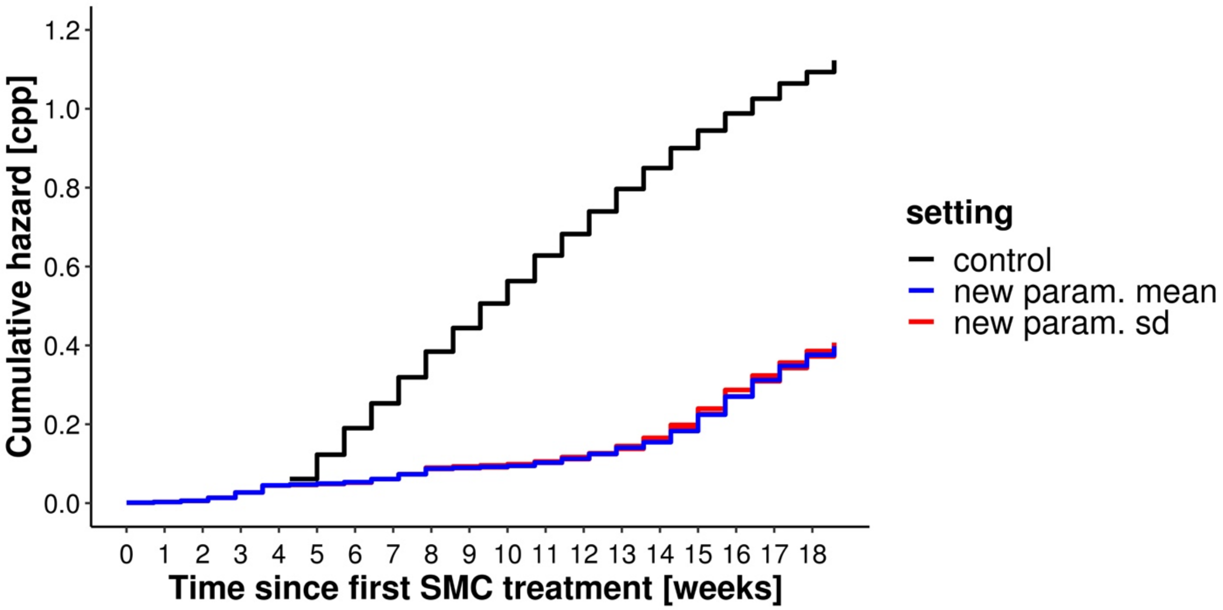

Fig M: Cumulative hazard of malaria in children who received SMC with SP-AQ (blue) compared to controls (black). The cumulative hazard over time was calculated using the Nelson-Aalen estimator. See Fig 2 in Zongo et al.(2015)[3] for comparison.

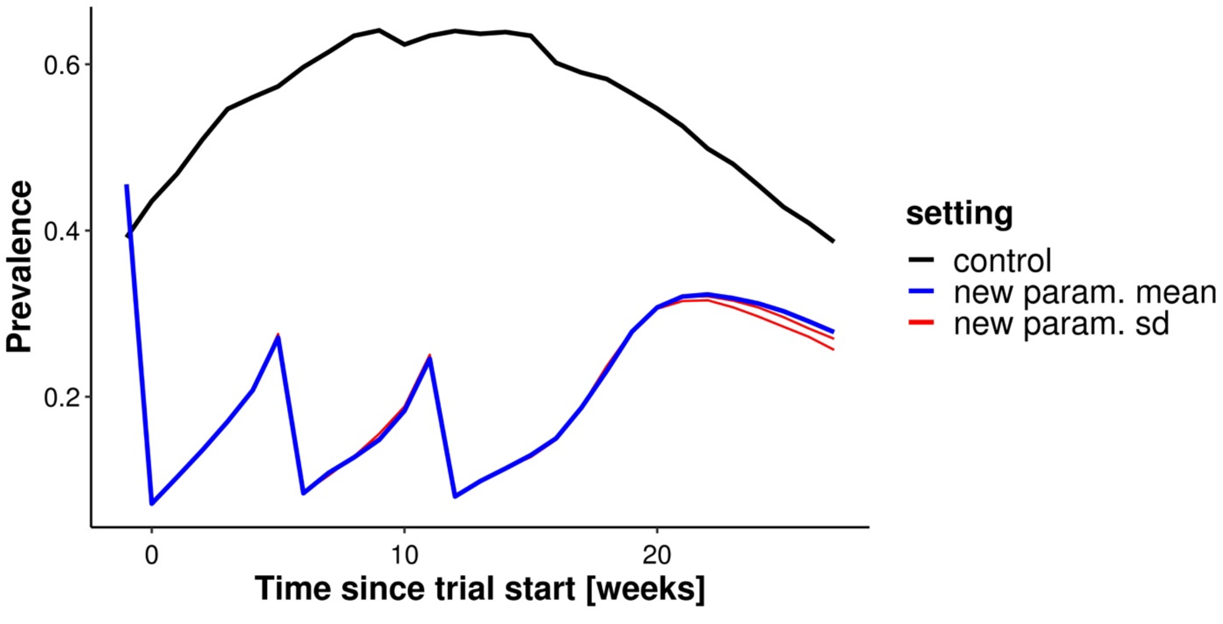

Fig N: Prevalence of malaria in children who received SMC with SP-AQ (blue) compared to controls (black) over the trial. The prevalence in the intervention cohort is decreasing with each SMC round every month and then slowly increasing again. At the same time, the prevalence in the control group is increasing with the ongoing malaria transmission season. After the last SMC round, there is an increase of prevalence caused by still ongoing transmission. Simultaneously, the transmission intensity is already decreasing as can be seen in the control cohort prevalence.

References

1. WHO. WHO Policy Recommendation: Seasonal Malaria Chemoprevention (SMC) for Plasmodium falciparum malaria control in highly seasonal transmission areas of the Sahel sub-region in Africa. (World Health Organization, 2012).

2. Dahal, P. et al. Statistical methods to derive efficacy estimates of anti-malarials for uncomplicated Plasmodium falciparum malaria: pitfalls and challenges. Malar. J. **16**, 430–430 (2017).

3. Zongo, I. et al. Randomized Noninferiority Trial of Dihydroartemisinin-Piperaquine Compared with Sulfadoxine-Pyrimethamine plus Amodiaquine for Seasonal Malaria Chemoprevention in Burkina Faso. Antimicrob. Agents Chemother. **59**, 4387–4396 (2015).

4. Dabire, K. R. et al. Anopheles funestus (Diptera: Culicidae) in a humid savannah area of western Burkina Faso: bionomics, insecticide resistance status, and role in malaria transmission. J Med Entomol **44**, 990–7 (2007).

5. WHO. World malaria report 2008. (World Health Organization, 2008).

6. Penny, M. A. et al. Distribution of malaria exposure in endemic countries in Africa considering country levels of effective treatment. Malar. J. **14**, 384 (2015).
